# Immune checkpoint inhibitors plus chemotherapy for early-stage or locally –advanced triple-negative breast cancer: a systematic review and Bayesian network meta-analysis of randomized trials

**DOI:** 10.3389/fonc.2026.1883161

**Published:** 2026-09-04

**Authors:** Ying Leng, Jiaqiang Dan, Qiuhan Heng, Junyan Li

**Affiliations:** The Fifth People’s Hospital of Chengdu, Chengdu, China

**Keywords:** Bayesian network meta-analysis, grade, immune checkpoint inhibitors, neoadjuvant therapy, pathological complete response, triple-negative breast cancer

## Abstract

**Background:**

Immune checkpoint inhibitors (ICIs) combined with chemotherapy have become a cornerstone in managing early and locally advanced triple-negative breast cancer (TNBC). Yet direct comparative evidence on the relative efficacy and safety of different PD-1/PD-L1 agents and treatment strategies—particularly neoadjuvant-only versus perioperative, “full-course” approaches—remains limited. To guide clinical decision-making, we performed a Bayesian network meta-analysis (NMA) comparing six specific ICI-based regimens and four overarching treatment strategies.

**Methods:**

We systematically searched PubMed, Embase, Cochrane Library, and Web of Science from database inception through February 25, 2026. Eligible randomized controlled trials (RCTs) enrolled patients with previously untreated early or locally advanced TNBC and compared ICI plus neoadjuvant chemotherapy with chemotherapy alone. The primary endpoint was pathological complete response (pCR); secondary endpoints included overall survival (OS) and grade ≥3 adverse events (AEs ≥3). We assessed risk of bias using the Cochrane RoB 2 tool. A Bayesian random-effects model was applied for the NMA; effects are reported as odds ratios (ORs) or hazard ratios (HRs) with 95% credible intervals (CrIs). Treatment rankings were derived from surface under the cumulative ranking curve (SUCRA) values. Certainty of evidence was evaluated using the GRADE framework, supplemented by the CINeMA tool.

**Results:**

Eight RCTs involving 2,073 patients met the inclusion criteria, encompassing four strategic treatment categories and six specific ICI-containing regimens. For pCR, PD-1 (neoadjuvant) + CT (OR 2.56, 95% CrI 1.55–4.22), PD-L1 (perioperative) + CT (OR 2.00, 95% CrI 1.29–3.09), and PD-1 (perioperative) + CT (OR 1.71, 95% CrI 1.39–2.10) each significantly improved pCR rates versus chemotherapy alone, though certainty of evidence ranged from moderate to very low. PD-L1 (neoadjuvant) + CT showed a numerical trend toward higher pCR (OR 1.41, 95% CrI 0.98–2.01) without reaching statistical significance. Regarding OS, both PD-L1 (neoadjuvant) + CT (HR 0.24, 95% CrI 0.08–0.72) and PD-1 (perioperative) + CT (HR 0.63, 95% CrI 0.48–0.82) demonstrated significant survival benefits over chemotherapy. In sensitivity analyses, durvalumab (neoadjuvant) + CT and pembrolizumab (perioperative) + CT yielded identical HRs (0.24 and 0.63, respectively). Safety profiles varied: camrelizumab (perioperative) + CT (OR 0.60, 95% CrI 0.34–1.04) and pembrolizumab (neoadjuvant) + CT (OR 0.53, 95% CrI 0.28–1.01) suggested reduced risks of AEs ≥3, but these findings did not achieve statistical significance and were supported by low-to-very-low certainty evidence.

**Conclusions:**

ICI–chemotherapy combinations consistently improve pCR rates in early and locally advanced TNBC. Notably, while our analysis hints at potential survival advantages with neoadjuvant PD-L1 blockade and perioperative PD-1 inhibition, these signals derive from only three studies and remain hypothesis-generating rather than practice-changing. Future head-to-head RCTs are warranted to confirm these observations and to better define long-term safety profiles.

**Systematic review registration:**

https://www.crd.york.ac.uk/prospero/, identifier CRD420261385223.

## 1 Introduction

Breast cancer remains the most frequently diagnosed malignancy among women worldwide and ranks fifth as a cause of cancer-related mortality. According to GLOBOCAN 2022 estimates, approximately 2.3 million new cases occur each year—accounting for 11.6% of all incident cancers—with roughly 670,000 deaths globally (1). Beyond its profound physiological and psychological toll on patients and families, the disease imposes substantial socioeconomic burdens through high treatment costs and lost workforce productivity. Triple-negative breast cancer (TNBC), defined by the absence of oestrogen receptor, progesterone receptor, and human epidermal growth factor receptor 2 expression, accounts for 15–20% of all breast cancers (2). Characterized by marked aggressiveness, early relapse, and limited targeted therapeutic options, TNBC carries the poorest prognosis among breast cancer subtypes (3).For decades, anthracycline- and taxane-based neoadjuvant chemotherapy represented the standard of care for patients with localized or operable TNBC. Nevertheless, pathological complete response (pCR) rates have remained suboptimal, typically ranging from 30% to 40% (4). Failure to achieve pCR is a strong predictor of poor long-term outcomes, including event-free and disease-free survival (5). Overcoming the efficacy ceiling of conventional chemotherapy to improve pCR rates—and, critically, to translate these gains into survival benefits—therefore represents a central clinical challenge in early-stage and locally advanced TNBC.

The advent of immune checkpoint inhibitors (ICIs) targeting the programmed death-1 (PD-1)/programmed death-ligand 1 (PD-L1) axis has reshaped the therapeutic landscape across solid tumours. The relatively high tumour mutational burden and dense tumour-infiltrating lymphocyte populations characteristic of TNBC provide a compelling biological rationale for immunotherapy (6). In the metastatic setting, randomised trials have shown that PD-1/PD-L1 inhibition combined with chemotherapy significantly prolongs progression-free and overall survival, paving the way for translation into earlier disease stages (7, 8). Landmark phase III trials—most notably KEYNOTE-522 and IMpassion031—established that adding ICIs to neoadjuvant chemotherapy increases pCR rates by an absolute 10 to 20 percentage points and improves event-free survival. These results have defined a new standard for high-risk early TNBC (9, 10).

Despite these advances, several unresolved questions persist. First, multiple ICIs with distinct molecular targets—anti-PD-1 versus anti-PD-L1 agents—are now clinically available, yet whether meaningful differences exist in their efficacy or safety profiles remains unclear (11). Second, the optimal duration and timing of ICI administration continue to provoke debate. The “perioperative, full-course” strategy exemplified by KEYNOTE-522 aims to eradicate minimal residual disease through extended immune exposure but raises concerns about prolonged treatment duration, cumulative toxicity, and cost. Conversely, the “neoadjuvant-only” approach investigated in trials such as GeparNuevo offers a shorter treatment course, but whether its long-term survival outcomes are non-inferior to the perioperative model remains unproven (12). Critically, no head-to-head randomised controlled trials (RCTs) have directly compared these agents or scheduling strategies.

Given this complexity, conventional pairwise meta-analyses cannot rank multiple interventions simultaneously. To fill this evidence gap, we performed a Bayesian network meta-analysis (NMA) synthesising all available RCT data. Our objective was to compare the efficacy and safety of specific ICIs (e.g., pembrolizumab, atezolizumab, camrelizumab) and overarching treatment strategies (perioperative versus neoadjuvant-only) across key endpoints—including pCR, overall survival, and grade ≥3 adverse events. By generating probabilistic treatment rankings, this analysis aims to provide high-level evidence to inform clinical decision-making and future guideline development.

## 2 Materials and methods

This network meta-analysis (NMA) was conducted in accordance with the Preferred Reporting Items for Systematic Reviews and Meta-Analyses extension statement for network meta-analyses (PRISMA-NMA; **Supplementary Table 1**) (13). To ensure transparency and mitigate outcome reporting bias, the study protocol was prospectively registered with PROSPERO (CRD420261385223).

### 2.1 Data sources and search strategy

We performed a systematic literature search across PubMed, EMBASE, the Cochrane Library, Web of Science, ClinicalTrials.gov, and the WHO International Clinical Trials Registry Platform (ICTRP). Grey literature was supplemented by hand-searching abstracts from major oncology conferences (SABCS, ASCO, ESMO) over the preceding five years, along with pharmaceutical company websites and relevant dissertations. The search strategy combined Medical Subject Headings (MeSH) and free-text terms related to triple-negative breast cancer (e.g., “Triple-Negative Breast Neoplasms,” “ER-Negative PR-Negative HER2-Negative Breast Cancer”), neoadjuvant therapy (e.g., “Neoadjuvant Therapies”), and immune checkpoint inhibitors (e.g., “Checkpoint Inhibitors, Immune,” “pembrolizumab,” “atezolizumab”) (**Supplementary Table 2**). Records were searched from database inception through February 25, 2026, with no language restrictions.

### 2.2 Eligibility criteria

Inclusion criteria were as follows: (1) randomised controlled trials (RCTs) enrolling adults with histologically confirmed, treatment-naïve, operable early-stage or locally advanced TNBC; (2) intervention arms comprising a PD-1 or PD-L1 inhibitor combined with standard neoadjuvant chemotherapy—both “neoadjuvant-only” and “perioperative (full-course)” strategies were eligible; (3) control arms receiving standard neoadjuvant chemotherapy alone; and (4) availability of at least one of the following outcomes—pathological complete response (pCR), overall survival (OS), or grade ≥3 adverse events (AEs ≥3). pCR was defined as the absence of invasive carcinoma in both breast and axilla per local pathology assessment. OS was defined as the time from randomisation to death from any cause. AEs ≥3 were graded according to the National Cancer Institute Common Terminology Criteria for Adverse Events (CTCAE).

Exclusion criteria were: (1) RCTs reporting on overlapping patient populations or duplicate publications; (2) studies with incomplete or inadequately reported outcome data; and (3) non-randomised studies, reviews, or case reports.

After removing duplicates, two reviewers independently screened all records by title and abstract. Full-text articles were then assessed for eligibility. Discrepancies during screening or data extraction were resolved through consensus or by consulting a third senior investigator. For each included trial, we ensured that the most recent and comprehensive dataset was extracted.

### 2.3 Data extraction and quality assessment

Using a standardised, pilot-tested form, two investigators independently extracted data from each eligible publication. Extracted variables included trial name, NCT identifier, first author, publication year, trial phase, sample size, median age, intervention and control regimens, and tumour stage. For efficacy and safety outcomes, we extracted odds ratios (ORs) with 95% credible intervals (CrIs) for pCR and AEs ≥3, and hazard ratios (HRs) with 95% CrIs for OS. For multi-arm platform trials such as I-SPY2, only data from TNBC-specific cohorts were included; HER2-positive and hormone receptor-positive subgroups were excluded.

Methodological quality was assessed using the Cochrane RoB 2 tool (14). This instrument evaluates bias across five domains: the randomisation process, deviations from intended interventions, missing outcome data, measurement of the outcome, and selection of the reported result. Each domain, along with the overall risk of bias, was categorised as “low risk,” “some concerns,” or “high risk.”

### 2.4 Statistical analysis

A Bayesian network meta-analysis (NMA) was performed using the rjags and gemtc packages in R (R Foundation for Statistical Computing, Vienna, Austria). Random-effects models served as the primary analytical framework to account for potential clinical and methodological heterogeneity across trials; fixed-effect models were fitted for comparison. Vague priors were assigned as follows: a normal distribution N(0, 10²) for the baseline log-odds ratio, and a uniform distribution U(0, 2) for the between-study heterogeneity variance (τ²). To test the robustness of these assumptions, sensitivity analyses employed an alternative half-Cauchy prior, Cauchy^+^(0, 2.5), for τ², evaluating stability in effect estimates and treatment rankings (SUCRA). Multi-arm randomised controlled trials (RCTs) were handled by splitting the shared control arm proportionally across active comparators to avoid double-counting of participants.

Model parameters were estimated via Markov Chain Monte Carlo (MCMC) simulation, initiating three independent chains. Each chain underwent a burn-in phase of 20,000 iterations, followed by 100,000 sampling iterations for posterior estimation. Convergence was assessed visually using trace and density plots, supplemented by the Gelman–Rubin diagnostic (potential scale reduction factor, PSRF); convergence was deemed adequate when PSRF approached 1.0.

Treatment rankings were derived from the posterior distributions using surface under the cumulative ranking curve (SUCRA) values; values closer to 1 indicated a higher probability of superior efficacy. For safety outcomes (AEs ≥3), SUCRA interpretation was inverted such that higher values reflected a lower risk of toxicity. Ranking probabilities were extracted via the rank.probabilityfunction and visualised as heatmaps using the pheatmap package to illustrate the distribution of treatments across rank positions.

Model fit was evaluated using residual deviance and the Deviance Information Criterion (DIC). A random-effects model was preferred if the DIC difference (ΔDIC) exceeded 3 compared with the fixed-effect model; ΔDIC < 3 suggested negligible differences in model performance. Global network consistency was appraised by comparing consistency and inconsistency models; a ΔDIC > 5 was considered indicative of meaningful inconsistency. All effect estimates are reported as odds ratios (ORs) with 95% credible intervals (CrIs).

Small-study effects and publication bias were assessed in STATA version 18.0 (StataCorp, College Station, TX, USA) using comparison-adjusted funnel plots. Given the limited interpretability of funnel plots in sparse networks, these analyses were restricted to outcomes with an adequate number of contributing studies and interpreted cautiously alongside study size, effect magnitude, and clinical heterogeneity.

### 2.5 Grading of evidence using GRADE and CINeMA

The certainty of evidence was evaluated using the GRADE/CINeMA framework.Randomised controlled trials (RCTs) were taken as the starting point for high-certainty evidence. Overall certainty was downgraded based on assessments across six domains: risk of bias, indirectness, imprecision, heterogeneity, incoherence, and publication bias or small-study effects.

Risk of bias was evaluated at the study level using the Cochrane RoB 2 tool. CINeMA’s contribution matrix was then used to weight and aggregate these risks according to the proportion of information each trial contributed to the specific network estimate. Indirectness was assessed by examining the transitivity and exchangeability assumptions. We prespecified potential effect modifiers—including baseline disease severity, intervention intensity, and follow-up duration—and compared the consistency of direct and indirect evidence with respect to population characteristics, interventions, comparators, and outcome measurements.

Imprecision was evaluated using prespecified thresholds for the minimal important difference (MID). For dichotomous outcomes, an odds ratio (OR) of 1.25 was defined as the threshold for a clinically important effect; evidence was rated down if the 95% credible interval (CrI) crossed both the null value (OR = 1.0) and this MID threshold. Heterogeneity was informed primarily by the between-study variance (τ²) estimated from the random-effects model, considered together with the location of the 95% prediction interval relative to the MID.

For networks containing closed loops, incoherence (inconsistency) was assessed using CINeMA’s built-in direct-versus-indirect evidence comparisons, applying either node-splitting or design-by-treatment interaction models. Publication bias and small-study effects were evaluated by triangulating trial registration status and grey literature searches with visual inspection of comparison-adjusted funnel plots.

Each domain was rated as “no concerns,” “some concerns,” or “serious concerns.” Following GRADE guidance, evidence certainty was downgraded by one level for domains with some concerns and by two levels for those with serious concerns. The final certainty ratings were categorised as high, moderate, low, or very low.

## 3 Results

### 3.1 Study selection and characteristics

**Figure 1:**
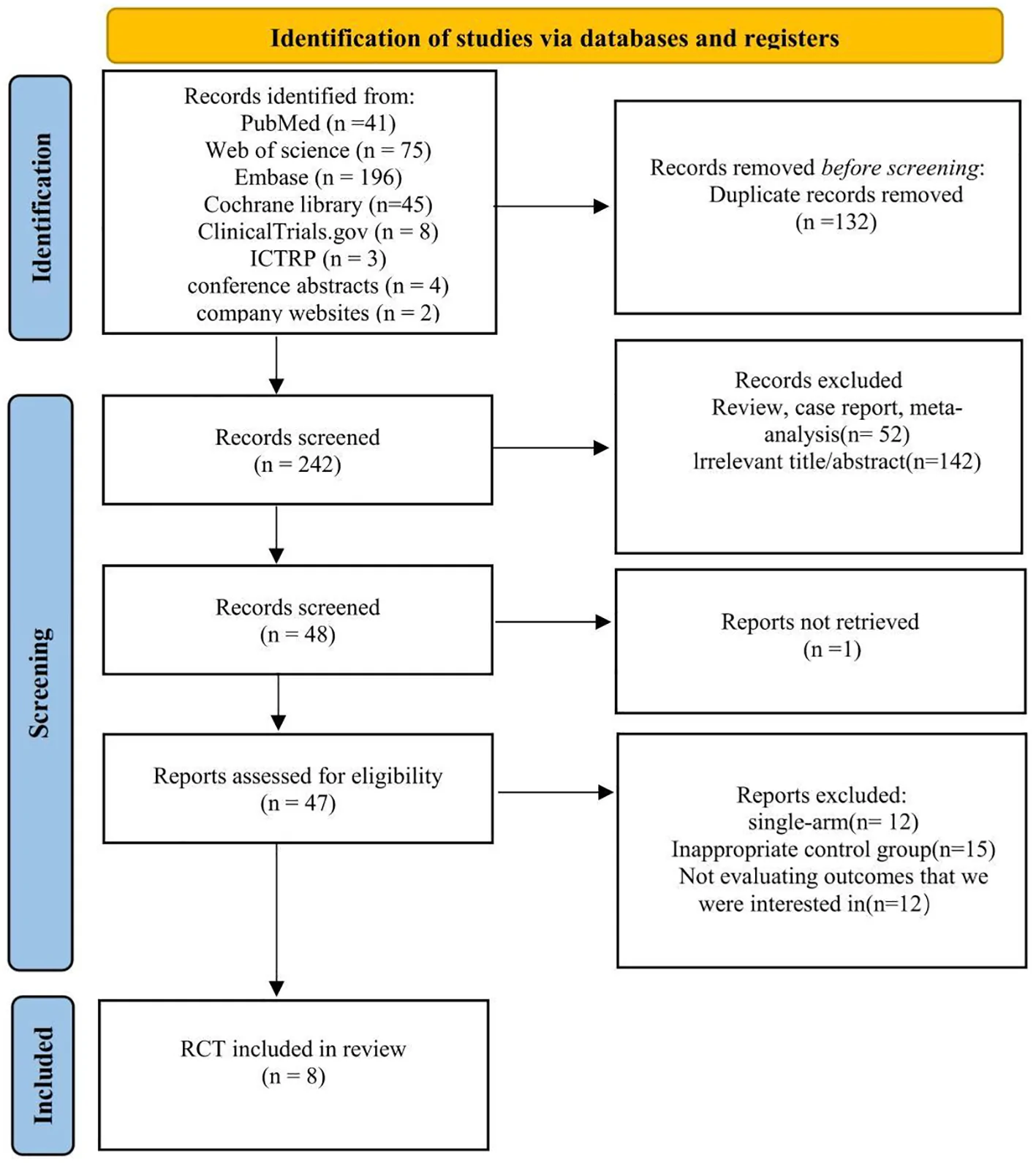
Flow diagram of the literature search and study selection process [this study was conducted in accordance with the PRISMA (preferred reporting items for systematic reviews and meta-analyses) guidelines].

The systematic search yielded 374 unique records. After title and abstract screening, 47 full-text articles were assessed for eligibility. Ultimately, eight randomised controlled trials (RCTs) met the inclusion criteria and were incorporated into the analysis (**Figure 1**) (15–22). These trials enrolled a total of 2,073 patients across six specific intervention arms: neoadjuvant pembrolizumab plus chemotherapy (Pembro-[NA]+CT), neoadjuvant atezolizumab plus chemotherapy (Atezo-[NA]+CT), neoadjuvant durvalumab plus chemotherapy (Durva-[NA]+CT), perioperative atezolizumab plus chemotherapy (Atezo-[P]+CT), perioperative camrelizumab plus chemotherapy (Camre-[P]+CT), and perioperative pembrolizumab plus chemotherapy (Pembro-[P]+CT). For analytical purposes, these regimens were further grouped into four overarching strategies: neoadjuvant PD-1 inhibition plus chemotherapy (PD-1[NA]+CT), neoadjuvant PD-L1 inhibition plus chemotherapy (PD-L1[NA]+CT), perioperative PD-1 inhibition plus chemotherapy (PD-1[P]+CT), and perioperative PD-L1 inhibition plus chemotherapy (PD-L1[P]+CT).

All included trials were RCTs comparing ICI-containing regimens against standard neoadjuvant chemotherapy alone. Core chemotherapeutic backbones consisted of taxane-, anthracycline-, and platinum-based agents. The study populations comprised patients with stage II–III early or locally advanced TNBC. Sample sizes ranged from several dozen to several hundred participants per trial. Methodological designs were evenly split, with four open-label and four double-blind studies; randomisation schemes were predominantly 1:1 (n = 5), followed by 1:4 (n = 1) and 2:1 (n = 2).

Data availability varied across endpoints. All eight trials provided pathological complete response (pCR) data. Overall survival (OS) data were available from three trials with mature follow-up, encompassing 1,123 patients. AEs ≥3 data were extracted from five trials involving 905 patients. Detailed characteristics of the included studies are summarised in **Table 1**.

**Table 1:** Baseline characteristics of studies included in the network meta-analysis.

Study (tear; phase)	Registered ID (NCT)	Randomization ratio	Sample size (Exp./Ctrl.)	Stage	ICI agent (timing)	pCR definition
I-SPY2 (2020; open)	NCT01042379	≈1:4	29/181	II–III	Pembrolizumab (NA only)	ypT0/Tis ypN0
GeparNuevo (2022; db)	NCT02685059	1:1	88/86	cT1b–cT4a	Durvalumab (NA only)	ypT0 ypN0
NCI-10013 (2022; open)	NCT02883062	2:1	45/16	II–III	Atezolizumab (NA only)	ypT0/Tis ypN0
NeoTRIP (2022; open)	NCT02620280	1:1	138/142	II–III	Atezolizumab (NA only)	ypT0 ypN0
PLANeT Trial (2025; open)	-	1:1	78/79	II–III	Pembrolizumab (NA only, low-dose)	ypT0 ypN0
IMpassion031 (2024; db)	NCT03197935	1:1	165/168	II–III	Atezolizumab (Peri)	ypT0/Tis ypN0
KEYNOTE-522 (2024; db)	NCT03036488	2:1	784/390	II–III	Pembrolizumab (Peri)	ypT0/Tis ypN0
CamRelief (2024; db)	NCT04613674	1:1	222/219	II–III	Camrelizumab (Peri)	ypT0/Tis ypN0

db, double-blind; NA, neoadjuvant only; Peri, perioperative (neoadj+adjuv); Pembro, pembrolizumab; Durva, durvalumab; Atezo, atezolizumab; Camre, camrelizumab; ICI, immune checkpoint inhibitor; pCR, pathological complete response.

### 3.2 Risk of bias assessment

**Figure 2:**
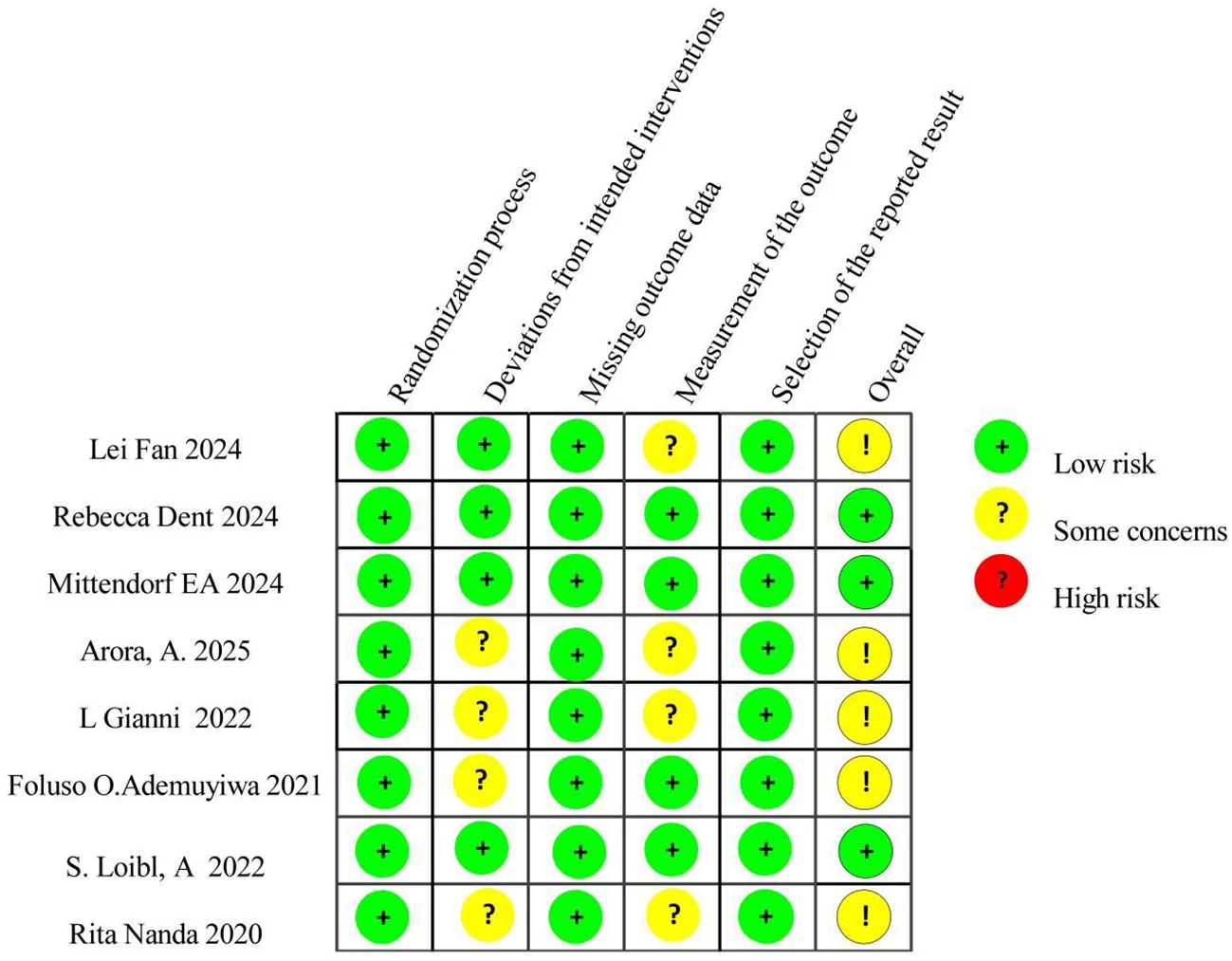
Summary of results from assessment of studies by using Cochrane risk of bias tool 2.0.

Risk of bias for the eight included RCTs was evaluated using the Cochrane RoB 2 tool (**Figure 2**). Overall, three trials were judged to be at low risk of bias, while five raised some concerns; no trial was deemed to be at high risk of bias, supporting a generally robust evidence base. At the domain level, all eight trials demonstrated low risk of bias regarding the randomisation process and the selection of reported results. Similarly, all trials were rated as low risk for missing outcome data. In contrast, only four trials were considered low risk with respect to deviations from intended interventions, and an equivalent number met the criteria for low risk of bias in outcome measurement.

### 3.3 Network meta-analysis by immunotherapy target class

#### 3.3.1 Overall survival and pathological complete response

**Figure 3:**
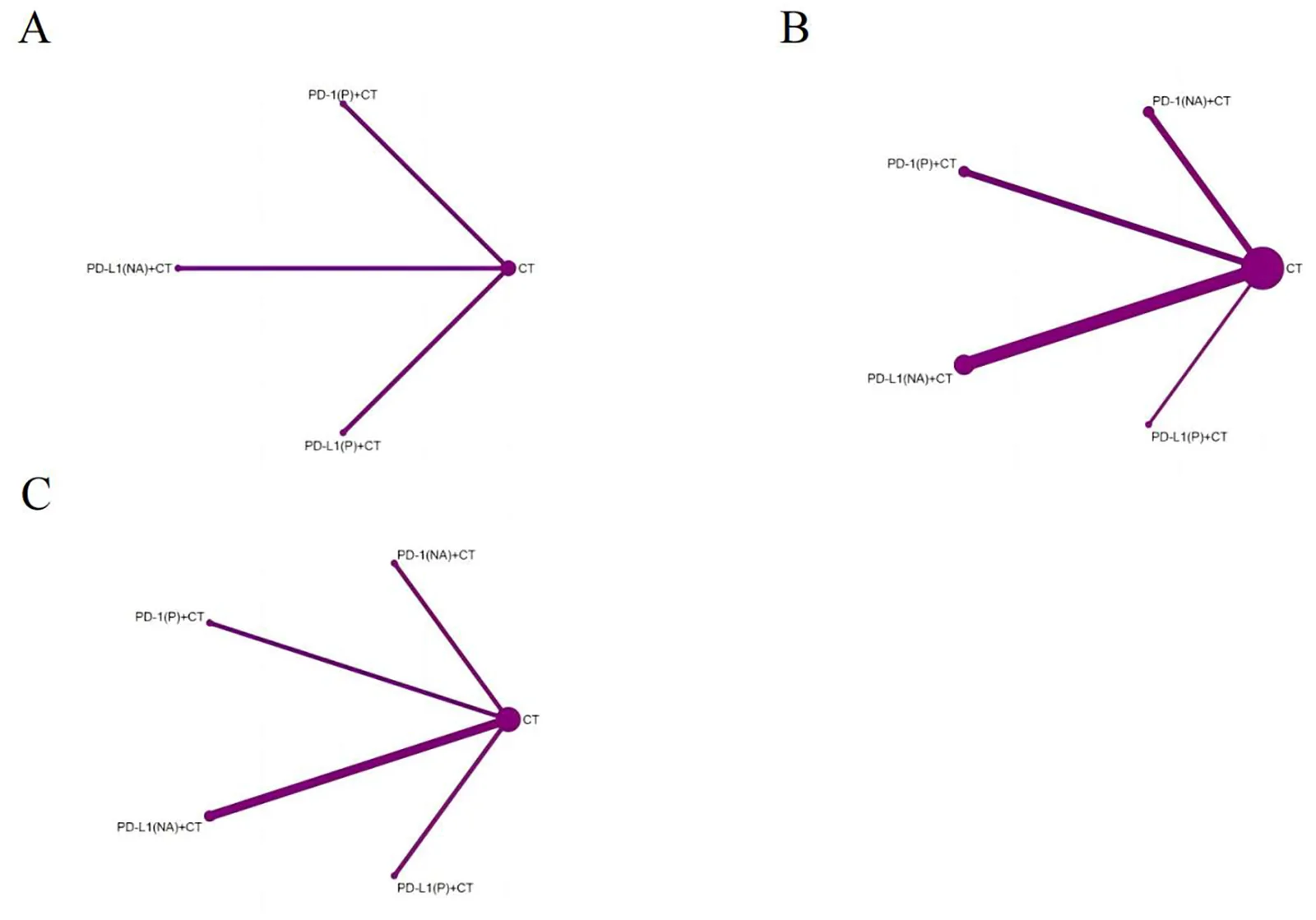
Network geometry for comparisons of immune checkpoint inhibitors by inhibitor class. **(A)** Network plot for overall survival (OS). **(B)** Network plot for pathological complete response (pCR). **(C)** Network plot for AEs ≥3.

**Figure 4:**
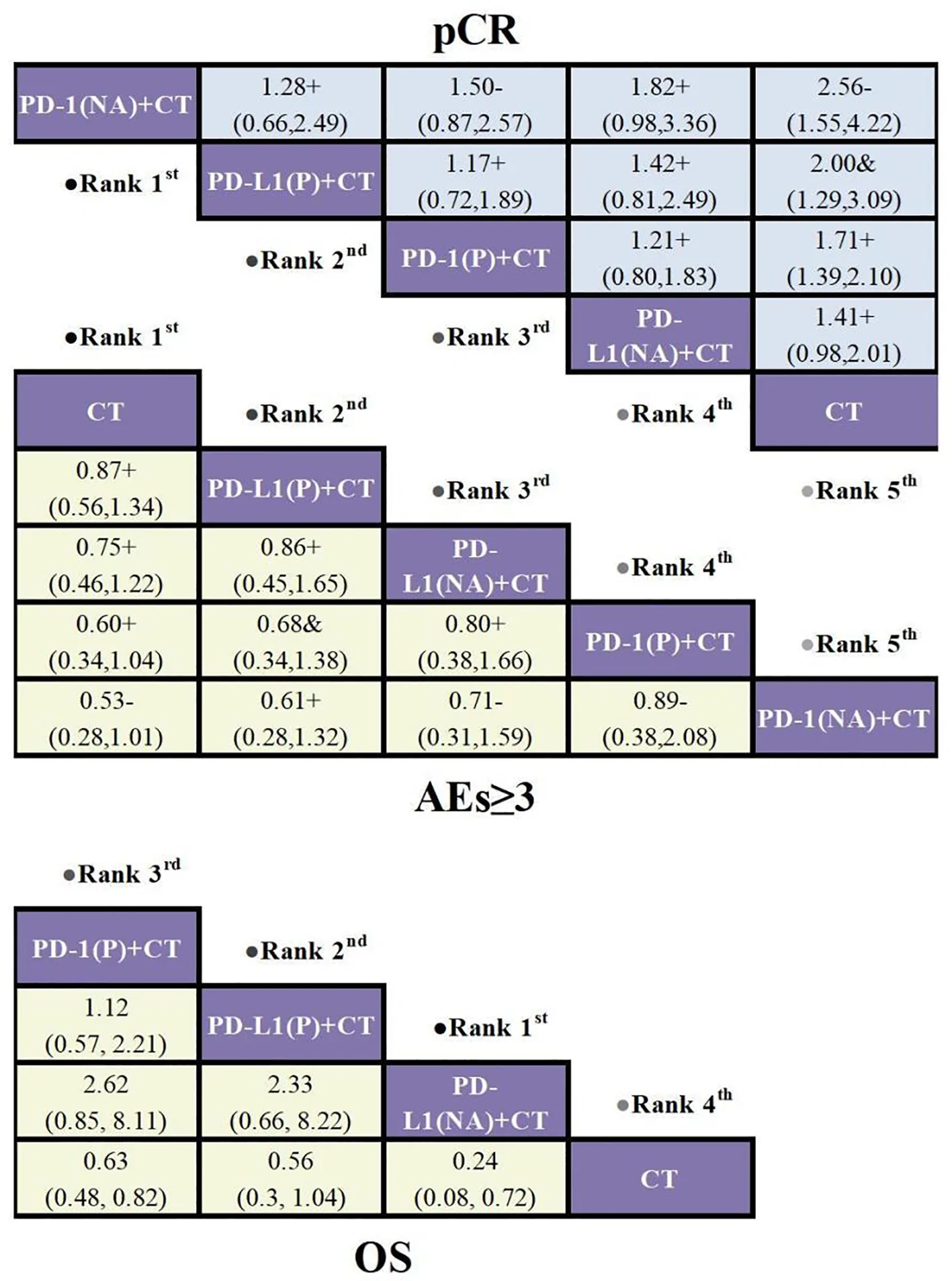
League tables and GRADE evidence profiles for immune checkpoint inhibitors by inhibitor class regarding OS, pCR, and AEs ≥3. Symbols indicate the certainty of evidence according to GRADE classification: *, high; &, moderate; +, low; -, very low.

For overall survival (OS), data from three trials encompassing 1,123 patients were pooled across three intervention arms (**Figure 3**). Compared with chemotherapy alone (CT), both neoadjuvant PD-L1 inhibition plus CT (PD-L1[NA]+CT; HR 0.24, 95% CrI 0.08–0.72) and perioperative PD-1 inhibition plus CT (PD-1[P]+CT; HR 0.63, 95% CrI 0.48–0.82) significantly improved survival outcomes in early TNBC (**Figure 4**).

For pathological complete response (pCR), eight trials involving 2,073 patients contributed data across four intervention strategies (**Figure 3**). Relative to CT, neoadjuvant PD-1 inhibition plus CT (PD-1[NA]+CT; OR 2.56, 95% CrI 1.55–4.22) was associated with a marked increase in pCR rates, albeit based on very low–certainty evidence. Perioperative PD-L1 inhibition plus CT (PD-L1[P]+CT; OR 2.00, 95% CrI 1.29–3.09) and perioperative PD-1 inhibition plus CT (PD-1[P]+CT; OR 1.71, 95% CrI 1.39–2.10) similarly demonstrated significant improvements in pCR, with certainty ranging from moderate to low. Although neoadjuvant PD-L1 inhibition plus CT (PD-L1[NA]+CT; OR 1.41, 95% CrI 0.98–2.01) suggested a numerical trend toward higher pCR rates, this effect did not reach statistical significance (**Figure 4**).

#### 3.3.2 Grade ≥3 adverse events

Five trials involving 905 patients provided data on AEs ≥3 across four intervention strategies (**Figure 3**). Based on low- to very low–certainty evidence, both perioperative PD-1 inhibition plus CT (PD-1[P]+CT; OR 0.60, 95% CrI 0.34–1.04) and neoadjuvant PD-1 inhibition plus CT (PD-1[NA]+CT; OR 0.53, 95% CrI 0.28–1.01) exhibited a trend toward reduced toxicity compared with CT; however, these differences were not statistically significant (**Figure 4**).

### 3.4 Network meta-analysis by specific immune checkpoint inhibitor agent

#### 3.4.1 Overall survival and pathological complete response

**Figure 5:**
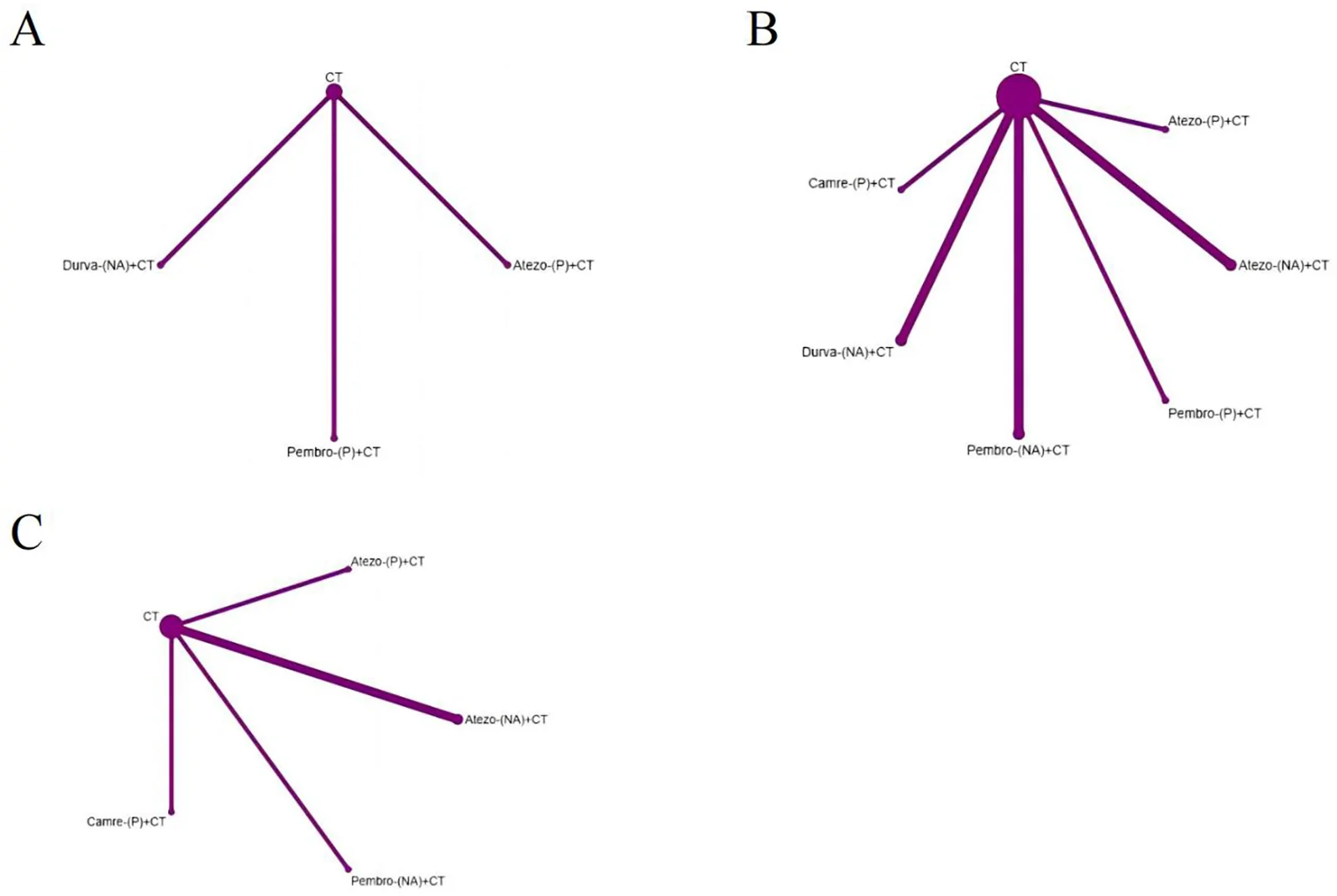
Network geometry for comparisons of specific immune checkpoint inhibitor agents. **(A)** Network plot for overall survival (OS). **(B)** Network plot for pathological complete response (pCR). **(C)** Network plot for AEs ≥3.

**Figure 6:**
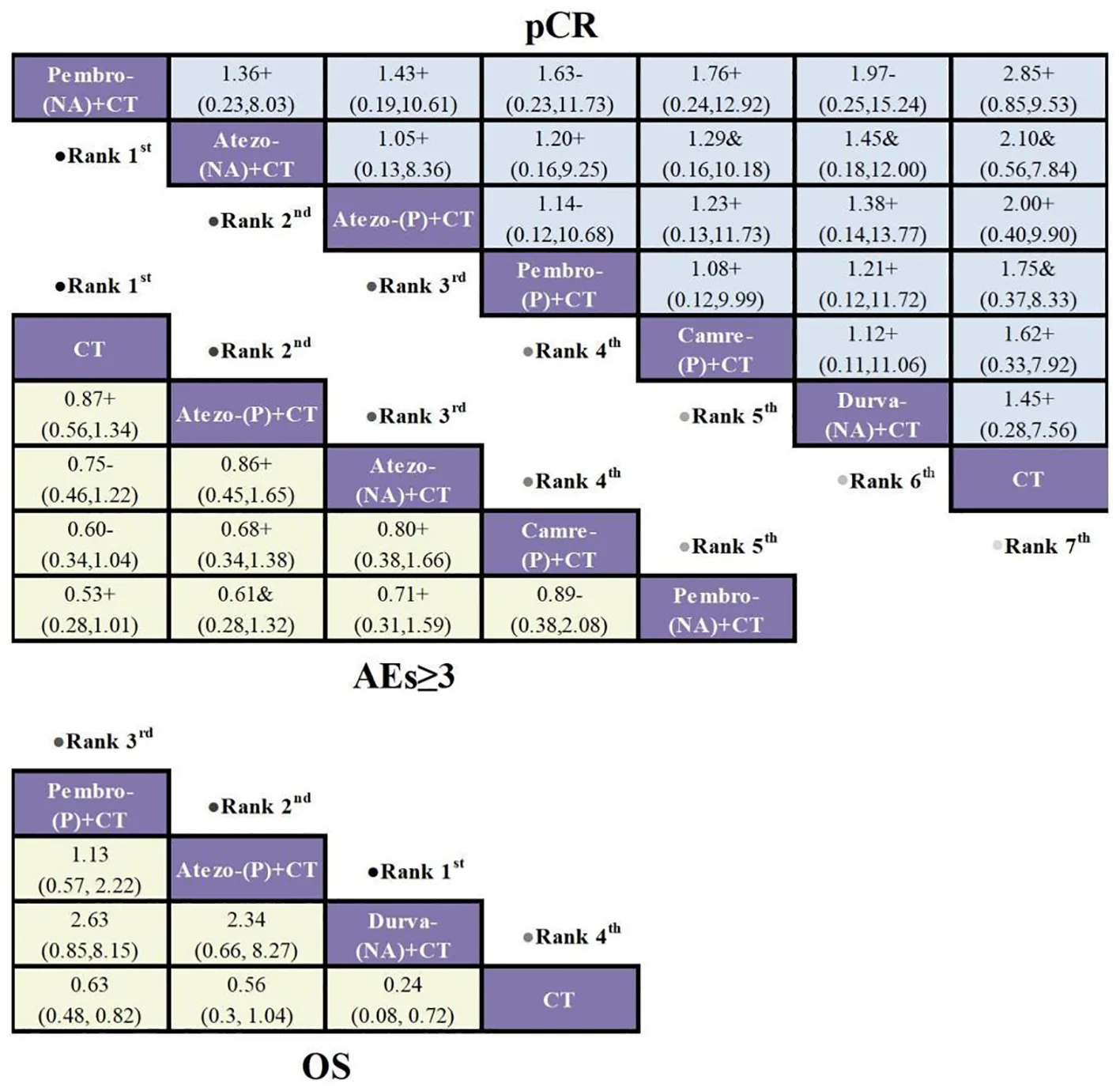
League tables and GRADE evidence profiles for specific immune checkpoint inhibitor agents regarding OS, pCR, and AEs ≥3. Symbols indicate the certainty of evidence according to GRADE classification: *, high; &, moderate; +, low; -, very low.

For overall survival (OS), three trials involving 1,123 patients were analysed across three specific regimens (**Figure 5**). Compared with chemotherapy alone (CT), both neoadjuvant durvalumab plus CT (Durva-[NA]+CT; HR 0.24, 95% CrI 0.08–0.72) and perioperative pembrolizumab plus CT (Pembro-[P]+CT; HR 0.63, 95% CrI 0.48–0.82) conferred significant survival benefits in early TNBC (**Figure 6**).

For pathological complete response (pCR), eight trials comprising 2,073 patients evaluated six specific ICI-containing regimens (**Figure 5**). Neoadjuvant pembrolizumab plus CT (Pembro-[NA]+CT; OR 2.85, 95% CrI 0.85–9.53) showed a numerical trend toward higher pCR rates relative to CT, but this association did not reach statistical significance (**Figure 6**).

#### 3.4.2 Grade ≥3 adverse events

Five trials with 905 patients contributed safety data across four specific regimens (**Figure 5**). Low- to very low–certainty evidence indicated that both neoadjuvant pembrolizumab plus CT (Pembro-[NA]+CT; OR 0.53, 95% CrI 0.28–1.01) and perioperative camrelizumab plus CT (Camre-[P]+CT; OR 0.60, 95% CrI 0.34–1.04) were associated with a trend toward lower rates of AEs ≥3 compared with CT; none of these differences reached statistical significance (**Figure 6**).

### 3.5 Treatment ranking

**Figure 7:**
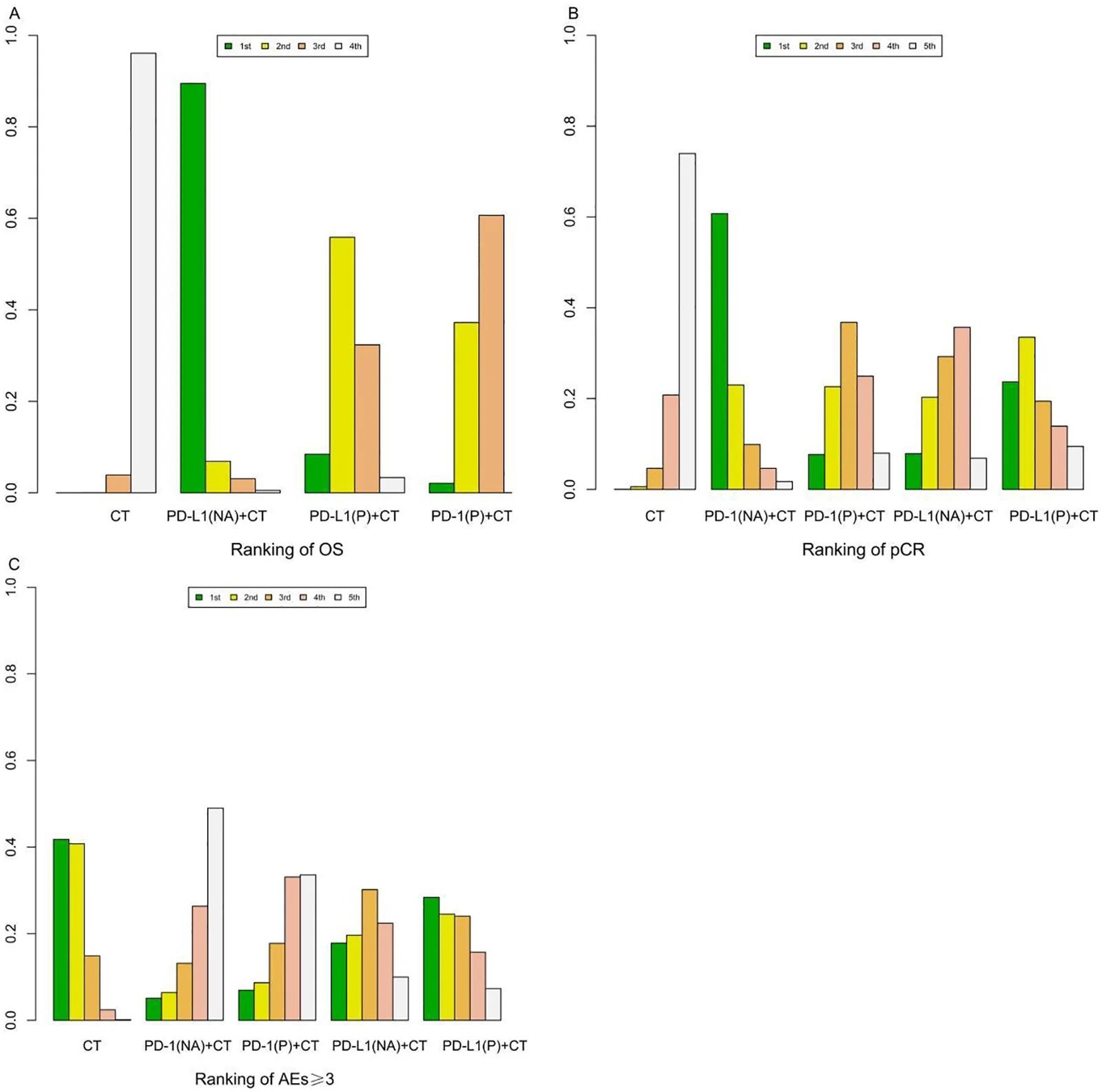
Ranking profiles for immune checkpoint inhibitors by inhibitor class regarding **(A)**​ OS, **(B)**​ pCR, and **(C)** AEs ≥3.

**Figure 8:**
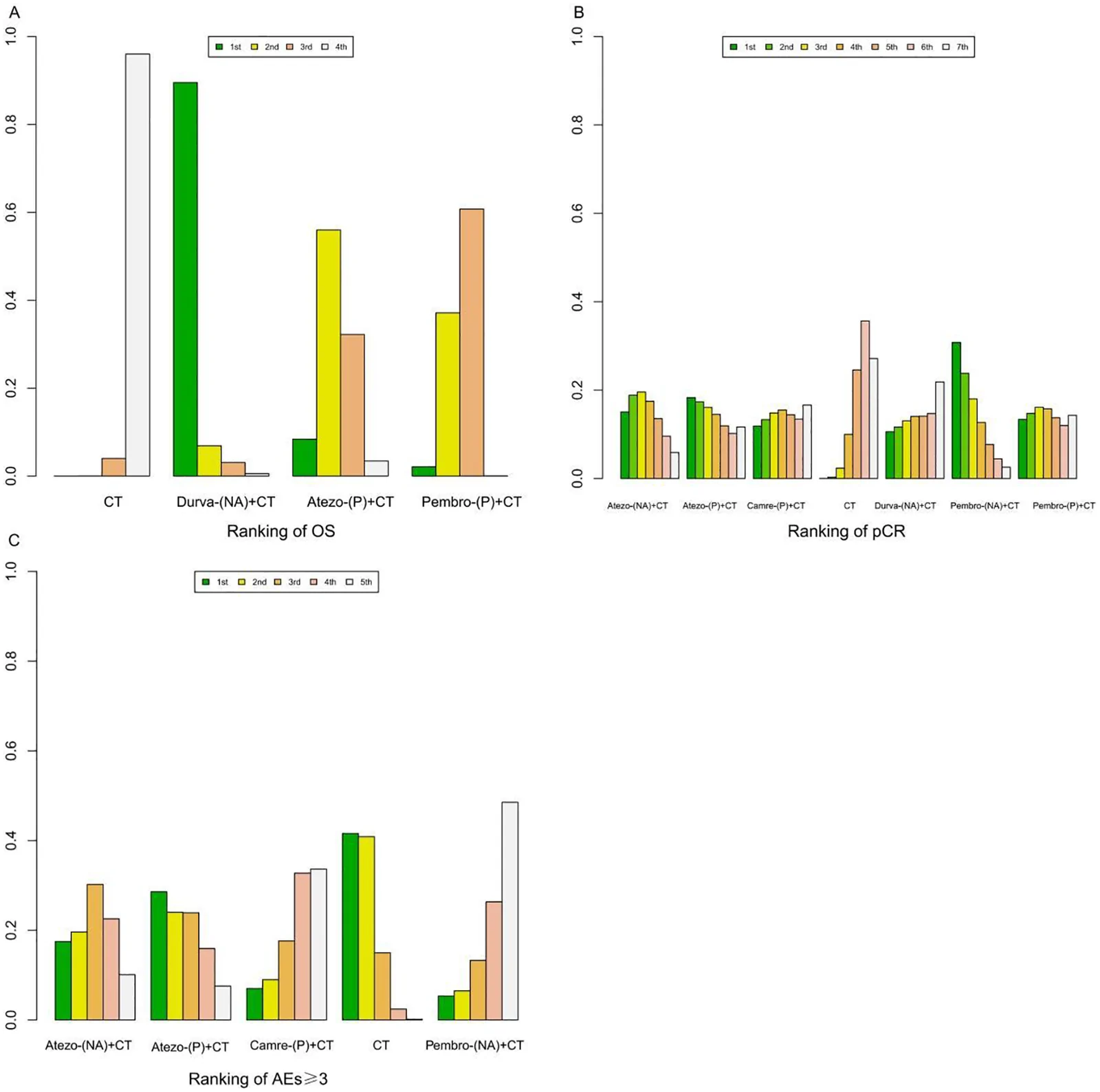
Ranking profiles for specific immune checkpoint inhibitor agents regarding **(A)**​ OS, **(B)**​ pCR, and **(C)**​ AEs ≥3.

Bayesian ranking based on surface under the cumulative ranking curve (SUCRA) values (**Figures 7**, **8**; **Supplementary Tables 7, 8** and **Supplementary Figures 13–18**) revealed distinct performance profiles across immunotherapy strategies in early and locally advanced TNBC. When stratified by drug class, neoadjuvant PD-L1 inhibition plus chemotherapy (PD-L1[NA]+CT) emerged as the most probable treatment for improving overall survival (OS), with a SUCRA of 95.1%. Among PD-1–based strategies, neoadjuvant PD-1 inhibition plus chemotherapy (PD-1[NA]+CT) ranked highest for achieving pathological complete response (pCR; SUCRA = 82.4%), underscoring its potential advantage in augmenting short-term treatment response. Perioperative PD-L1 inhibition plus chemotherapy (PD-L1[P]+CT) occupied the second position for pCR (SUCRA = 56.4%).

At the individual agent level, neoadjuvant durvalumab plus chemotherapy (Durva-[NA]+CT) was the regimen most likely to optimise OS (probability of ranking first = 95.1%). Neoadjuvant pembrolizumab plus chemotherapy (Pembro-[NA]+CT) held the highest probability of achieving pCR (probability of ranking first = 72.2%). With respect to safety, chemotherapy alone ranked first for minimising the risk of AEs ≥3 (SUCRA = 80.3%).These rankings are exploratory in nature. Because SUCRA values are derived from indirect comparisons, their reliability depends on the quality of the underlying evidence. Accordingly, these results should not be construed as definitive guidance for clinical decision-making.

### 3.6 Sensitivity analyses and assessment of bias

Convergence of the Markov Chain Monte Carlo (MCMC) simulations was evaluated using diagnostic plots and the Gelman–Rubin statistic. Visual inspection of trace and density plots (**Supplementary Figures 1–12**) indicated stable mixing across all chains, satisfying standard convergence criteria. Global network consistency was assessed by comparing the deviance information criterion (DIC) between consistency and inconsistency models. As shown in **Supplementary Tables 9**, **10**, the absolute DIC difference (ΔDIC) was less than 3 for all outcomes, indicating no evidence of meaningful inconsistency and supporting the assumption of transitivity across the evidence network.

Between-study heterogeneity was low across outcomes, with I² values ranging from 6% to 34%. Model selection favoured random-effects models; however, the ΔDIC between random- and fixed-effect specifications was consistently below 3 (**Supplementary Tables 11**, **12**), suggesting that effect estimates were robust to the choice of modelling framework. To evaluate the influence of low-dose pembrolizumab regimens, we conducted a sensitivity analysis excluding the PLANeT trial. The re-estimated network did not alter the geometry of the treatment network for pathological complete response (pCR) or grade ≥3 adverse events (AEs ≥3). Crucially, point estimates and 95% credible intervals (CrIs) for all interventions remained materially unchanged (**Supplementary Figure 21**).

Given the sparsity of the evidence base (fewer than 10 studies per outcome), formal assessment of small-study effects was restricted to pCR, the only outcome informed by eight RCTs. Visual inspection of comparison-adjusted funnel plots revealed no obvious asymmetry. However, the low number of contributing studies limits the power to detect publication bias; thus, these findings should be interpreted with caution.

## 4 Discussion

### 4.1 Principal findings

The therapeutic landscape for early and locally advanced triple-negative breast cancer (TNBC) has broadened considerably, yet high-quality head-to-head comparisons remain scarce. By integrating direct and indirect evidence through a Bayesian network meta-analysis (NMA), this study offers a comparative assessment of the relative efficacy—measured by pathological complete response (pCR) and overall survival (OS)—and safety across various immune checkpoint inhibitor (ICI) regimens and scheduling strategies. That said, the limitations inherent to the available evidence must be acknowledged: OS analyses drew on just three trials, and safety data (AEs ≥3) came from five. These constraints call for measured interpretation of the findings.

With respect to survival, neoadjuvant PD-L1 inhibition plus chemotherapy (PD-L1[NA]+CT; specifically, durvalumab plus chemotherapy [Durva-[NA]+CT]) significantly reduced the risk of death compared with chemotherapy alone. Perioperative PD-1 inhibition plus chemotherapy (PD-1[P]+CT; specifically, pembrolizumab plus chemotherapy [Pembro-[P]+CT]) likewise conferred a significant survival advantage. These findings were corroborated by the treatment rankings: PD-L1[NA]+CT achieved a surface under the cumulative ranking curve (SUCRA) value of 95.1%, placing it first among all strategies, and Durva-[NA]+CT held the highest SUCRA (95.1%) among individual drug regimens.

For pCR, neoadjuvant PD-1 inhibition plus chemotherapy (PD-1[NA]+CT), perioperative PD-L1 inhibition plus chemotherapy (PD-L1[P]+CT), and perioperative PD-1 inhibition plus chemotherapy (PD-1[P]+CT) each significantly improved pCR rates relative to chemotherapy alone, though the certainty of evidence ranged from moderate to very low. Within these categories, PD-1[NA]+CT attained a SUCRA of 82.4%, and the specific regimen of neoadjuvant pembrolizumab plus chemotherapy (Pembro-[NA]+CT) reached a SUCRA of 72.2%, ranking first within their respective groupings.

Safety analyses, pooling data from five trials involving 905 patients, indicated that chemotherapy alone carried the lowest risk of AEs ≥3 (SUCRA = 80.3%), ranking first among all interventions. Notably, low- to very low–certainty evidence suggested that perioperative camrelizumab plus chemotherapy (Camre-[P]+CT; OR 0.60, 95% CrI 0.34–1.04) and neoadjuvant pembrolizumab plus chemotherapy (Pembro-[NA]+CT; OR 0.53, 95% CrI 0.28–1.01) trended toward lower toxicity compared with chemotherapy; however, these differences did not reach statistical significance.

The SUCRA rankings presented here reflect the relative performance of interventions within the current evidence network and are inherently exploratory. They are meant to generate hypotheses rather than to confirm the superiority of any particular agent or treatment duration, and they do not warrant changes in clinical practice. Additionally, event-free survival (EFS)—a more mature efficacy endpoint—was not included in the primary NMA owing to extreme data sparsity. Of the eight included trials, only KEYNOTE-522 and IMpassion031 reported EFS outcomes; while the former demonstrated a clear survival benefit, the latter showed a non-significant trend with wide confidence intervals. Given the heterogeneity between these two effect estimates and the limited sample size, we chose not to pool EFS data to avoid model instability and potential bias.

### 4.2 Interpretation of findings

Neoadjuvant immunotherapy refers to the integration of immune-modulating agents into conventional preoperative systemic therapy) (23). Broadly defined, cancer immunotherapy aims to recalibrate host immunity to elicit an endogenous antitumour response capable of eradicating malignant cells (24). Under homeostatic conditions, the immune system identifies and eliminates tumour cells within the microenvironment. However, malignancies such as triple-negative breast cancer (TNBC) frequently co-opt immune checkpoints—notably programmed death-ligand 1 (PD-L1)—to orchestrate immune evasion. PD-L1 engages its receptor, programmed death-1 (PD-1), primarily expressed on activated T cells. This ligation initiates signalling cascades that attenuate T-cell effector functions and dampen B-cell responses to tumour-associated antigens) (25, 26). Consequently, monoclonal antibodies targeting the PD-1/PD-L1 axis constitute the principal immunotherapeutic strategy in current clinical practice. Preclinical and translational data suggest that TNBC may be particularly susceptible to immune intervention, owing to its comparatively higher densities of tumour-infiltrating lymphocytes and frequent PD-L1 overexpression relative to other breast cancer subtypes (27, 28).

The biological rationale for neoadjuvant immunotherapy centres on exploiting the preoperative “window of opportunity,” when the antigenic repertoire is most intact and immunogenicity is maximal (29). An undisturbed primary tumour provides a rich source of neoantigens capable of priming a broad and potent systemic immune response (30). A systemically primed immune milieu not only targets the primary lesion but also facilitates the clearance of occult micrometastases beyond the resolution of conventional imaging—potentially mitigating long-term recurrence risk at its origin. In contrast, adjuvant immunotherapy administered postoperatively encounters a markedly reduced tumour burden and attenuated antigenic stimulation. Moreover, the physiologic stress of surgery may induce a transient state of immunosuppression, potentially blunting the efficiency and breadth of any subsequent immune activation (31). Thus, the preoperative setting represents the optimal timeframe to harness the full therapeutic potential of immunotherapy.

Synergy between chemotherapy and immune checkpoint inhibitors (ICIs) arises from complementary mechanisms. Conventional cytotoxic agents—such as taxanes and anthracyclines—exert direct tumouricidal effects while concurrently modulating the immune landscape. Chemotherapy can induce immunogenic cell death, resulting in the release of tumour-associated antigens and danger-associated molecular patterns (32–34). Additionally, it may deplete immunosuppressive cellular subsets within the tumour microenvironment and upregulate major histocompatibility complex class I (MHC-I) expression on tumour cells (35, 36). Collectively, these alterations reprogramme the tumour microenvironment from an immunosuppressive to an g state (37). Against this backdrop, PD-1/PD-L1 inhibitors effectively reverse T-cell exhaustion, restoring effector functions. This combinatorial approach aligns “chemotherapy-driven tumour debulking and antigen release” with “immunotherapy-mediated liberation of antitumour immunity,” yielding synergistic enhancements in pathological complete response (pCR) rates, as substantiated in pivotal clinical trials (9, 38).

In the present network meta-analysis, overall survival (OS) data were sufficiently mature in only three RCTs. Our findings indicate that neoadjuvant PD-L1 inhibition plus chemotherapy and perioperative PD-1 inhibition plus chemotherapy significantly reduced the risk of death compared with chemotherapy alone. The survival advantage observed with PD-L1 blockade may relate to preservation of PD-L2 signalling pathways and rapid reversal of T-cell exhaustion, whereas the benefits associated with perioperative PD-1 inhibition likely stem from sustained immune exposure, facilitating the eradication of minimal residual disease (9, 39). We emphasise that these mechanistic interpretations are derived from indirect comparisons and biological plausibility; given the limited OS evidence base, these conclusions remain hypothesis-generating and require validation in prospective head-to-head trials.

The discordance between markedly improved pCR rates and the as-yet immature OS signal can be attributed to three interrelated factors. First, pCR serves as an early pathological endpoint assessed shortly after therapy completion, whereas OS requires extended follow-up; most pivotal trials have not yet reached their predefined OS analysis timepoints (39). Second, while pCR correlates strongly with event-free and overall survival, it remains an imperfect surrogate. A subset of patients attaining pCR ultimately experience recurrence, whereas others who do not achieve pCR may attain durable remission through subsequent adjuvant therapies. Thus, increased pCR rates are strongly suggestive of, but not equivalent to, definitive OS gains (4). Finally, OS is confounded by post-recurrence therapies, including multiple lines of salvage treatment, which may attenuate or obscure initial differences in neoadjuvant outcomes, rendering survival signals statistically elusive (40).

Safety profiles in early-stage TNBC reflect the superimposition of chemotherapy-related toxicities and immune-mediated adverse events (irAEs) (41). PD-1/PD-L1 inhibitors unleash effector T-cell activity, which, while targeting neoplasia, may also precipitate autoimmunity manifesting as pneumonitis, colitis, hepatitis, or endocrinopathies. When combined with cytotoxic agents, these irAEs may coincide with chemotherapy-induced myelosuppression, neuropathy, and other class-specific toxicities. Data from key trials indicate that while AEs ≥3occur more frequently in absolute terms with combination therapy, this increase often lacks statistical significance when managed within structured monitoring and supportive care frameworks, suggesting an acceptable risk-benefit profile (9, 10, 39). Nevertheless, clinicians must remain vigilant for the distinct toxicity spectrum of immunotherapy and adhere to standardised management algorithms (42, 43). Crucially, while our analysis identified a numerical trend toward lower AEs ≥3 in certain ICI–chemotherapy regimens, all credible intervals crossed the null; thus, these regimens should not be construed as safer than chemotherapy alone. This inference is further tempered by heterogeneity across trials in adverse event reporting windows, chemotherapeutic backbones, and eligibility criteria, compounded by the limited sample size (five trials; n = 905), which elevates susceptibility to small-study biases. Importantly, our analysis did not disaggregate irAEs, which differ fundamentally from conventional chemotherapy toxicities in timing, pathophysiology, and management. Accordingly, clinicians should maintain a high index of suspicion for cumulative toxicities and rigorously implement irAE monitoring protocols.

### 4.3 Implications for clinical practice and future research

This network meta-analysis offers a tiered framework to inform neoadjuvant immunotherapy decisions in early and locally advanced TNBC. In clinical practice, incorporating PD-1 or PD-L1 inhibitors into standard neoadjuvant chemotherapy represents a reasonable approach for patients seeking to maximise pathological complete response (pCR) rates—whether to facilitate surgical downstaging or to improve long-term prognosis. Regarding safety, although the incidence of AEs ≥3 did not differ significantly between combination regimens and chemotherapy alone, the available evidence—constrained by limited sample size and imprecision—does not permit a definitive conclusion of non-inferiority. Clinicians should therefore remain alert to the possibility of additive toxicities, particularly in patients with comorbidities or compromised organ function.

Future research should prioritise four key directions. First, mature survival data are essential. Longer follow-up is needed to establish whether the observed pCR gains translate into durable improvements in overall survival (OS) and event-free survival (EFS). Second, head-to-head randomised trials comparing distinct immune checkpoint inhibitors (ICIs) or different scheduling strategies—for example, neoadjuvant-only versus perioperative regimens—are urgently required. Such phase III studies would provide the highest level of evidence to resolve current uncertainties surrounding optimal agent selection and treatment duration. Third, biomarker-driven subgroup analyses must be embedded into trial designs. Identifying predictive signatures would allow delineation of patient subsets most likely to benefit from ICI-based combinations, advancing precision oncology while sparing non-responders unnecessary toxicity and cost. Finally, comprehensive evaluations of long-term toxicities—including endocrine, cardiac, and pulmonary adverse events—together with health-related quality of life (HRQoL) measures are needed. Particular attention should be paid to characterising differential toxicity profiles between PD-1 and PD-L1 inhibitors, as subtle pharmacological differences may translate into clinically meaningful divergences in safety outcomes.

### 4.4 Comparison with prior evidence

To our knowledge, this is the first network meta-analysis to systematically compare the relative efficacy and safety of distinct immune checkpoint inhibitor classes—PD-1 versus PD-L1 inhibitors—and scheduling strategies—perioperative versus neoadjuvant-only—specifically in early and locally advanced triple-negative breast cancer (TNBC).

Our findings align with those of Villacampa et al., who recently reported in *JAMA Oncology* that neoadjuvant ICI-based therapy significantly improves outcomes in breast cancer (44). Their analysis, however, included a heterogeneous population spanning TNBC, hormone receptor-positive/HER2-negative disease, and HER2-positive breast cancer. By restricting our cohort exclusively to early and locally advanced TNBC—a biologically homogeneous entity—we effectively mitigated confounding arising from intrinsic biological differences and variable treatment responsiveness across breast cancer subtypes. Moreover, whereas Villacampa et al. applied conventional pairwise meta-analytic methods focused on pathological complete response (pCR) and event-free survival (EFS), our Bayesian NMA framework incorporated overall survival (OS) and grade ≥3 adverse events (AEs ≥3). This methodological advance moves the discussion beyond mere efficacy validation toward comparative effectiveness research, enabling direct and indirect comparisons between specific agents and scheduling strategies. Such granular evidence is not attainable through traditional meta-analytic approaches and provides higher-resolution data to inform precision clinical decision-making in TNBC.

Our results also concur with Petrelli et al. (45). in identifying ICI–chemotherapy combinations as the optimal strategy for maximising pCR rates relative to chemotherapy alone. Petrelli and colleagues, however, did not differentiate between the pharmacological mechanisms of specific ICIs or account for variations in treatment duration. Our analysis builds on this foundation by disentangling these variables and rigorously evaluating safety profiles alongside the certainty of evidence using GRADE/CINeMA frameworks. This layered approach yields a more granular risk–benefit assessment, filling critical gaps left by earlier syntheses.

### 4.5 Strengths and limitations

This study has several methodological and clinical strengths. First, we adhered strictly to a prospectively registered protocol and followed the PRISMA-NMA reporting guidelines. Rigorous procedures—including systematic literature searches, dual-reviewer screening, node-splitting to accommodate multi-arm trials, and random-effects modelling—ensured analytic robustness. Second, we applied the CINeMA framework to grade the certainty of evidence and interpreted primary outcomes against predefined minimal important difference thresholds. This approach prioritises clinically meaningful comparisons over statistical significance alone. Most notably, this NMA introduces a novel structural decomposition of interventions. By distinguishing between PD-1 and PD-L1 inhibitors and, critically, modelling “perioperative” versus “neoadjuvant-only” strategies as distinct nodes, we reduced conceptual heterogeneity. This enabled independent assessment of specific immunotherapeutic regimens, substantially improving the precision and clinical applicability of our findings.

Several limitations merit careful consideration. Although this NMA facilitated indirect comparisons between PD-1 and PD-L1 inhibitors, the validity of the transitivity assumption may be undermined by imbalances in key effect modifiers across trials. These include differences in chemotherapeutic backbones, varying degrees of PD-L1 enrichment among enrolled populations, and disparities in follow-up maturity. Furthermore, direct evidence comparing PD-1 versus PD-L1 agents remains scarce; consequently, these specific comparisons rely heavily on indirect estimates, the reliability of which hinges on the transitivity assumptions noted above. Regarding survival outcomes, OS data were available from only three RCTs. The limited number of studies and events resulted in an overall low certainty of evidence for OS. Finally, assessment of small-study effects for pCR was constrained by the number of contributing trials (n = 8), raising the possibility that unpublished small studies with null findings may not have been captured.

### 4.6 Conclusions

Immune checkpoint inhibitor–chemotherapy combinations consistently improve pathological complete response rates in early and locally advanced triple-negative breast cancer. While our analysis hints at potential survival advantages with neoadjuvant PD-L1 blockade plus chemotherapy and perioperative PD-1 inhibition, these signals derive from only three studies and remain hypothesis-generating rather than practice-changing. Future head-to-head randomised controlled trials are warranted to confirm these observations and to better define long-term safety profiles.

## Data Availability

Publicly available datasets were analyzed in this study. This data can be found here: Data extracted from published articles; see References and **Supplementary Material**.
