# Supplementary material for: Immune checkpoint inhibitors plus chemotherapy for early-stage or locally –advanced triple-negative breast cancer: a systematic review and Bayesian network meta-analysis of randomized trials
Source: Front Oncol. 2026 Sep 4;16:1883161. doi: 10.3389/fonc.2026.1883161 (PMC13585616; doi:10.3389/fonc.2026.1883161)
Supplement: Supplementary file 1 [file DataSheet1.docx]

| **Table of Contents** | | |
| --- | --- | --- |
| Title | Content | page |
| Table S1 | PRISMA NMA Checklist of Items to Include When Reporting a Systematic Review Involving a Network Meta-analysis | 2-8 |
| Table S2 | Literature Search Strategy | 9-14 |
| Table S3 | Baseline Characteristics of Studies Included in the Network Meta-Analysis | 15-17 |
| Table S4 | Pairwise Meta-Analysis of Pathological Complete Response: Individual Study Data and Pooled Odds Ratios | 18 |
| Table S5 | Pairwise Meta-Analysis of Overall Survival: Individual Study Hazard Ratios | 18 |
| Table S6 | Pairwise Meta-Analysis of Grade ≥3 Adverse Events: Individual Study Data and Pooled Odds Ratios | 19 |
| Table S7 | SUCRA-Based Ranking of Treatment Regimens for Endpoints in the Bayesian Network Meta-Analysis(Analysis by Target Class) | 19 |
| Table S8 | SUCRA-Based Ranking of Treatment Regimens for Endpoints in the Bayesian Network Meta-Analysis(Analysis by Specific Agent) | 20 |
| Table S9 | Model Fit Statistics for Consistency and Inconsistency Models in Bayesian Network Meta-Analysis by Target Class | 20 |
| Table S10 | Model Fit Statistics for Consistency and Inconsistency Models in Bayesian Network Meta-Analysis by Specific Agent | 21 |
| Table S11 | Model Fit Statistics for Fixed‑Effect and Random‑Effect Bayesian Network Meta‑Analysis by Target Class | 21 |
| Table S12 | Model Fit Statistics for Fixed‑Effect and Random‑Effect Bayesian Network Meta‑Analysis by Specific Agent | 22 |
| Table S13 | CINeMA (Confidence in Network Meta-Analysis) assessment of pathological complete response (pCR) for comparisons between PD-1 and PD-L1 inhibitors combined with chemotherapy versus chemotherapy alone or versus each other. | 23-24 |
| Table S14 | CINeMA assessment of adverse events (AEs) for class-level comparisons between PD-1 and PD-L1 inhibitors combined with chemotherapy versus chemotherapy alone or versus each other. | 25 |
| Table S15 | CINeMA assessment of pathological complete response (pCR) for agent-specific comparisons of immune checkpoint inhibitors combined with chemotherapy versus chemotherapy alone or versus each other. | 26-27 |
| Table S16 | CINeMA assessment of adverse events (AEs) for agent-specific comparisons of immune checkpoint inhibitors combined with chemotherapy versus chemotherapy alone or versus each other. | 28 |
| Figure S1 | MCMC Convergence Diagnostics for Overall Survival Stratified by Target Class. | 29 |
| Figure S2 | MCMC Convergence Diagnostics for Overall Survival Stratified by Specific Agent. | 30 |
| Figure S3 | MCMC Convergence Diagnostics for Pathological Complete Response Stratified by Target Class. | 31 |
| Figure S4 | MCMC Convergence Diagnostics for Pathological Complete Response Stratified by Specific Agent. | 32 |
| Figure S5 | MCMC Convergence Diagnostics for Grade ≥3 Adverse Events Stratified by Target Class. | 33 |
| Figure S6 | MCMC Convergence Diagnostics for Grade ≥3 Adverse Events Stratified by Specific Agent. | 34 |
| Figure S7 | Trajectory Curves and Density Plots of Overall Survival According to Immune Checkpoint Inhibitor Target Class. | 35 |
| Figure S8 | Trajectory Curves and Density Plots of Overall Survival According to Specific Immune Checkpoint Inhibitor Agents. | 36 |
| Figure S9 | Trajectory Curves and Density Plots of Pathological Complete Response According to Immune Checkpoint Inhibitor Target Class. | 37 |
| Figure S10 | Trajectory Curves and Density Plots of Pathological Complete Response According to Specific Immune Checkpoint Inhibitor Agents. | 38 |
| Figure S11 | Trajectory Curves and Density Plots of Grade ≥3 Adverse Events According to Immune Checkpoint Inhibitor Target Class. | 39 |
| Figure S12 | Trajectory Curves and Density Plots of Grade ≥3 Adverse Events According to Specific Immune Checkpoint Inhibitor Agents. | 40 |
| Figure S13 | Rank Probability Heatmap of Overall Survival Outcomes Stratified by Target Class. | 41 |
| Figure S14 | Rank Probability Heatmap of Overall Survival Outcomes Stratified by Specific Agent. | 42 |
| Figure S15 | Rank Probability Heatmap of Pathological Complete Response Outcomes Stratified by Target Class. | 43 |
| Figure S16 | Rank Probability Heatmap of Pathological Complete Response Outcomes Stratified by Specific Agent. | 44 |
| Figure S17 | Rank Probability Heatmap of Grade ≥3 Adverse Events Outcomes Stratified by Target Class . | 45 |
| Figure S18 | Rank Probability Heatmap of Grade ≥3 Adverse Events Outcomes Stratified by Specific Agent. | 46 |
| Figure S19 | Funnel Plots Illustrating Pathological Complete Response Rates According to Immune Checkpoint Inhibitor Target Class. | 47 |
| Figure S20 | Funnel Plots Illustrating Pathological Complete Response Rates According to Specific Immune Checkpoint Inhibitor Agents. | 48 |
| Figure S21 | Sensitivity Analysis League Table for Pathological Complete Response and Grade ≥3 Adverse Events (Excluding PLANeT Trial). | 49 |

| **Table S1. PRISMA NMA Checklist of Items to Include When Reporting a Systematic Review Involving a Network Meta-analysis** | | | |
| --- | --- | --- | --- |
| Section/Topic | Item # | Checklist Item | Page # |
| TITLE |  |  |  |
| Title | 1 | Identify the report as a systematic review incorporating  anetwork meta-analysis (or related form of meta-analysis). | 1 |
|  |  |  |  |
| ABSTRACT |  |  |  |
| Structured summary | 2 | Provide a structured summary including, as applicable:  **Background**: main objectives  **Methods**: data sources; study eligibility criteria, participants, and interventions; study appraisal; and synthesis methods, such as network meta-analysis.  **Results**: number of studies and participants identified; summary estimates with corresponding confidence/credible intervals; treatment rankings may also be discussed. Authors may choose to summarize pairwise comparisons against a chosen treatment included in their analyses for brevity.  **Discussion/Conclusions**: limitations; conclusions and implications of findings.  **Other**: systematic review registration number with registry name. | 1 |
|  |  |  |  |
| INTRODUCTION |  |  |  |
| Rationale | 3 | Describe the rationale for the review in the context of what is already known, including mention of why a network meta-analysis has been conducted. | 2 |
| Objectives | 4 | Provide an explicit statement of questions being addressed, with reference to participants, interventions, comparisons, outcomes, and study design (PICOS). | 2 |
|  |  |  |  |
| METHODS |  |  |  |
| Protocol and registration | 5 | Indicate whether a review protocol exists and if and where it can be accessed (e.g., Web address); and, if available, provide registration information, including registration number. | 3 |
| Eligibility criteria | 6 | Specify study characteristics (e.g., PICOS, length of follow-up) and report characteristics (e.g., years considered, language, publication status) used as criteria for eligibility, giving rationale. Clearly describe eligible treatments included in the treatment network, and note whether any have been clustered or merged into the same node (with justification). | 3 |
| Information sources | 7 | Describe all information sources (e.g., databases with dates of coverage, contact with study authors to identify additional studies) in the search and date last searched. | 3 |
| Search | 8 | Present full electronic search strategy for at least one database, including any limits used, such that it could be repeated. | 3，Supplementary  TableS2 |
| Study selection | 9 | State the process for selecting studies (i.e., screening, eligibility, included in systematic review, and, if applicable, included in the meta-analysis). | 3，Table 1 |
| Data collection process | 10 | Describe method of data extraction from reports (e.g., piloted forms, independently, in duplicate) and any processes for obtaining and confirming data from investigators. | 3 |
| Data items | 11 | List and define all variables for which data were sought (e.g., PICOS, funding sources) and any assumptions and simplifications made. | 3 |
| Geometry of the network | S1 | Describe methods used to explore the geometry of the treatment network under study and potential biases related to it. This should include how the evidence base has been graphically summarized for presentation, and what characteristics were compiled and used to describe the evidence base to readers. | 3 |
| Risk of bias within individual studies | 12 | Describe methods used for assessing risk of bias of individual studies (including specification of whether this was done at the study or outcome level), and how this information is to be used in any data synthesis. | 3 |
| Summary measures | 13 | State the principal summary measures (e.g., risk ratio, difference in means). Also describe the use of additional summary measures assessed, such as treatment rankings and surface under the cumulative ranking curve (SUCRA) values, as well as modified approaches used to present summary findings from meta-analyses. | 3 |
| Planned methods of analysis | 14 | Describe the methods of handling data and combining results of studies for each network meta-analysis. This should include, but not be limited to:   - Handling of multi-arm trials; - Selection of variance structure; - Selection of prior distributions in Bayesian analyses; and - Assessment of model fit. | 3-4 |
| Assessment of Inconsistency | S2 | Describe the statistical methods used to evaluate the agreement of direct and indirect evidence in the treatment network(s) studied. Describe efforts taken to address its presence when found. | 4 |
| Risk of bias across studies | 15 | Specify any assessment of risk of bias that may affect the cumulative evidence (e.g., publication bias, selective reporting within studies). | 4 |
| Additional analyses | 16 | Describe methods of additional analyses if done, indicating which were pre-specified. This may include, but not be limited to, the following:   - Sensitivity or subgroup analyses; - Meta-regression analyses; - Alternative formulations of the treatment network; and - Use of alternative prior distributions for Bayesian analyses (if applicable). | 4 |
| RESULTS† |  |  |  |
| Study selection | 17 | Give numbers of studies screened, assessed for eligibility, and included in the review, with reasons for exclusions at each stage, ideally with a flow diagram. | 4，Fig1 |
| Presentation of network structure | S3 | Provide a network graph of the included studies to enable visualization of the geometry of the treatment network. | 18,20，Fig3,5 |
| Summary of network geometry | S4 | Provide a brief overview of characteristics of the treatment network. This may include commentary on the abundance of trials and randomized patients for the different interventions and pairwise comparisons in the network, gaps of evidence in the treatment network, and potential biases reflected by the network structure. | 18,20 |
| Study characteristics | 18 | For each study, present characteristics for which data were extracted (e.g., study size, PICOS, follow-up period) and provide the citations. | 4,Table 1 |
| Risk of bias within studies | 19 | Present data on risk of bias of each study and, if available, any outcome level assessment. | 4,Figure2 |
| Results of individual studies | 20 | For all outcomes considered (benefits or harms), present, for each study: 1) simple summary data for each intervention group, and 2) effect estimates and confidence intervals. Modified approaches may be needed to deal with information from larger networks. | 5,Figure5-6 |
| Synthesis of results | 21 | Present results of each meta-analysis done, including confidence/credible intervals. In larger networks, authors may focus on comparisons versus a particular comparator (e.g. placebo or standard care), with full findings presented in an appendix. League tables and forest plots may be considered to summarize pairwise comparisons. If additional summary measures were explored (such as treatment rankings), these should also be presented. | 5-6 Figure 7,8, Supplementary Tables S3,S4, Figure S13–S18 |
| Exploration for inconsistency | S5 | Describe results from investigations of inconsistency. This may include such information as measures of model fit to compare consistency and inconsistency models, P values from statistical tests, or summary of inconsistency estimates from different parts of the treatment network. | 6,Supplementary Table S5–S6 |
| Risk of bias across studies | 22 | Present results of any assessment of risk of bias across studies for the evidence base being studied. | 6,Supplementary Tables S7,S8 |
| Results of additional analyses | 23 | Give results of additional analyses, if done (e.g., sensitivity or subgroup analyses, meta-regression analyses, alternative network geometries studied, alternative choice of prior distributions for Bayesian analyses, and so forth). | Supplementary Figure S1–S12 |
|  |  |  |  |
| DISCUSSION |  |  |  |
| Summary of evidence | 24 | Summarize the main findings, including the strength of evidence for each main outcome; consider their relevance to key groups (e.g., healthcare providers, users, and policy-makers). | 23-24 |
| Limitations | 25 | Discuss limitations at study and outcome level (e.g., risk of bias), and at review level (e.g., incomplete retrieval of identified research, reporting bias). Comment on the validity of the assumptions, such as transitivity and consistency. Comment on any concerns regarding network geometry (e.g., avoidance of certain comparisons). | 6-9 |
| Conclusions | 26 | Provide a general interpretation of the results in the context of other evidence, and implications for future research. | 9 |
|  |  |  |  |
| FUNDING |  |  |  |
| Funding | 27 | Describe sources of funding for the systematic review and other support (e.g., supply of data); role of funders for the systematic review. This should also include information regarding whether funding has been received from manufacturers of treatments in the network and/or whether some of the authors are content experts with professional conflicts of interest that could affect use of treatments in the network. | NA |

PICOS = population, intervention, comparators, outcomes, study design.

* Text in italics indicate S wording specific to reporting of network meta-analyses that has been added to guidance from the PRISMA statement.

† Authors may wish to plan for use of appendices to present all relevant information in full detail for items in this section.

| **Table S2.Literature Search Strategy** | |
| --- | --- |
| **Pubmed** | #1 "Triple Negative Breast Neoplasms"[MeSH Terms] OR ("er negative pr negative her2 negative breast cancer"[Title/Abstract] OR "er negative pr negative her2 negative breast cancer"[Title/Abstract] OR (("ER-Negative"[All Fields] AND "PR-Negative"[All Fields]) AND "her2 negative breast neoplasms"[Title/Abstract]) OR ((("eye rep"[Journal] OR "expert rev mol med"[Journal] OR "eng regen"[Journal] OR "educ res"[Journal] OR "econ rec"[Journal] OR "ER"[All Fields]) AND ("Negative"[All Fields] OR "negatively"[All Fields] OR "negatives"[All Fields] OR "negativities"[All Fields] OR "negativity"[All Fields]) AND ("psychopathol rev"[Journal] OR "pharmacol rep"[Journal] OR "pharmacognosy res"[Journal] OR "partis rev"[Journal] OR "PR"[All Fields]) AND ("Negative"[All Fields] OR "negatively"[All Fields] OR "negatives"[All Fields] OR "negativities"[All Fields] OR "negativity"[All Fields])) AND "her2 negative breast neoplasms"[Title/Abstract]) OR "triple negative breast cancer"[Title/Abstract] OR "triple negative breast cancer"[Title/Abstract] OR "breast cancers triple negative"[Title/Abstract] OR "breast cancer triple negative"[Title/Abstract] OR "triple negative breast cancers"[Title/Abstract] OR "triple negative breast neoplasm"[Title/Abstract] OR "breast neoplasms triple negative"[Title/Abstract] OR (("breast neoplasms"[MeSH Terms] OR ("Breast"[All Fields] AND "Neoplasms"[All Fields]) OR "breast neoplasms"[All Fields] OR ("Breast"[All Fields] AND "Neoplasm"[All Fields]) OR "breast neoplasm"[All Fields]) AND "Triple-Negative"[Title/Abstract]) OR "triple negative breast neoplasm"[Title/Abstract] OR "Triple Negative Breast Neoplasms"[Title/Abstract]) 29638  #2 "Neoadjuvant Therapy"[MeSH Terms] OR ("neoadjuvant therapies"[Title/Abstract] OR "therapy neoadjuvant"[Title/Abstract] OR "neoadjuvant treatment"[Title/Abstract] OR "neoadjuvant treatments"[Title/Abstract] OR "treatment neoadjuvant"[Title/Abstract] OR "neoadjuvant chemotherapy"[Title/Abstract] OR "chemotherapy neoadjuvant"[Title/Abstract] OR "neoadjuvant chemotherapies"[Title/Abstract] OR "neoadjuvant chemotherapy treatment"[Title/Abstract] OR (("chemotherapy s"[All Fields] OR "drug therapy"[MeSH Terms] OR ("drug"[All Fields] AND "Therapy"[All Fields]) OR "drug therapy"[All Fields] OR "Chemotherapies"[All Fields] OR "drug therapy"[MeSH Subheading] OR "Chemotherapy"[All Fields]) AND "treatment neoadjuvant"[Title/Abstract]) OR "neoadjuvant chemotherapy treatments"[Title/Abstract] OR "treatment neoadjuvant chemotherapy"[Title/Abstract] OR "neoadjuvant chemoradiotherapy"[Title/Abstract] OR "chemoradiotherapy neoadjuvant"[Title/Abstract] OR "neoadjuvant chemoradiotherapies"[Title/Abstract] OR "neoadjuvant chemoradiation"[Title/Abstract] OR "chemoradiation neoadjuvant"[Title/Abstract] OR (("neoadjuvancy"[All Fields] OR "Neoadjuvant Therapy"[MeSH Terms] OR ("Neoadjuvant"[All Fields] AND "Therapy"[All Fields]) OR "Neoadjuvant Therapy"[All Fields] OR "Neoadjuvant"[All Fields] OR "neoadjuvants"[All Fields] OR "neoadjuvent"[All Fields]) AND "Chemoradiations"[Title/Abstract]) OR "neoadjuvant chemoradiation therapy"[Title/Abstract] OR "chemoradiation therapy neoadjuvant"[Title/Abstract] OR "neoadjuvant chemoradiation therapies"[Title/Abstract] OR "therapy neoadjuvant chemoradiation"[Title/Abstract] OR "neoadjuvant chemoradiation treatment"[Title/Abstract] OR (("Chemoradiotherapy"[MeSH Terms] OR "Chemoradiotherapy"[All Fields] OR "Chemoradiation"[All Fields]) AND "treatment neoadjuvant"[Title/Abstract]) OR (("neoadjuvancy"[All Fields] OR "Neoadjuvant Therapy"[MeSH Terms] OR ("Neoadjuvant"[All Fields] AND "Therapy"[All Fields]) OR "Neoadjuvant Therapy"[All Fields] OR "Neoadjuvant"[All Fields] OR "neoadjuvants"[All Fields] OR "neoadjuvent"[All Fields]) AND "chemoradiation treatments"[Title/Abstract]) OR "treatment neoadjuvant chemoradiation"[Title/Abstract] OR "neoadjuvant systemic therapy"[Title/Abstract] OR "neoadjuvant systemic therapies"[Title/Abstract] OR "systemic therapy neoadjuvant"[Title/Abstract] OR "therapy neoadjuvant systemic"[Title/Abstract] OR "neoadjuvant systemic treatment"[Title/Abstract] OR "neoadjuvant systemic treatments"[Title/Abstract] OR "systemic treatment neoadjuvant"[Title/Abstract] OR (("therapeutics"[MeSH Terms] OR "therapeutics"[All Fields] OR "Treatments"[All Fields] OR "Therapy"[MeSH Subheading] OR "Therapy"[All Fields] OR "Treatment"[All Fields] OR "treatment s"[All Fields]) AND "neoadjuvant systemic"[Title/Abstract]) OR "neoadjuvant radiotherapy"[Title/Abstract] OR (("neoadjuvancy"[All Fields] OR "Neoadjuvant Therapy"[MeSH Terms] OR ("Neoadjuvant"[All Fields] AND "Therapy"[All Fields]) OR "Neoadjuvant Therapy"[All Fields] OR "Neoadjuvant"[All Fields] OR "neoadjuvants"[All Fields] OR "neoadjuvent"[All Fields]) AND "Radiotherapies"[Title/Abstract]) OR "radiotherapy neoadjuvant"[Title/Abstract] OR "neoadjuvant radiation"[Title/Abstract] OR (("neoadjuvancy"[All Fields] OR "Neoadjuvant Therapy"[MeSH Terms] OR ("Neoadjuvant"[All Fields] AND "Therapy"[All Fields]) OR "Neoadjuvant Therapy"[All Fields] OR "Neoadjuvant"[All Fields] OR "neoadjuvants"[All Fields] OR "neoadjuvent"[All Fields]) AND "Radiations"[Title/Abstract]) OR "radiation neoadjuvant"[Title/Abstract] OR "neoadjuvant radiation therapy"[Title/Abstract] OR (("neoadjuvancy"[All Fields] OR "Neoadjuvant Therapy"[MeSH Terms] OR ("Neoadjuvant"[All Fields] AND "Therapy"[All Fields]) OR "Neoadjuvant Therapy"[All Fields] OR "Neoadjuvant"[All Fields] OR "neoadjuvants"[All Fields] OR "neoadjuvent"[All Fields]) AND "radiation therapies"[Title/Abstract]) OR "radiation therapy neoadjuvant"[Title/Abstract] OR (("therapeutics"[MeSH Terms] OR "therapeutics"[All Fields] OR "Therapies"[All Fields] OR "Therapy"[MeSH Subheading] OR "Therapy"[All Fields] OR "therapy s"[All Fields] OR "therapys"[All Fields]) AND "neoadjuvant radiation"[Title/Abstract]) OR "neoadjuvant radiation treatment"[Title/Abstract] OR (("neoadjuvancy"[All Fields] OR "Neoadjuvant Therapy"[MeSH Terms] OR ("Neoadjuvant"[All Fields] AND "Therapy"[All Fields]) OR "Neoadjuvant Therapy"[All Fields] OR "Neoadjuvant"[All Fields] OR "neoadjuvants"[All Fields] OR "neoadjuvent"[All Fields]) AND "radiation treatments"[Title/Abstract]) OR "radiation treatment neoadjuvant"[Title/Abstract] OR (("therapeutics"[MeSH Terms] OR "therapeutics"[All Fields] OR "Treatments"[All Fields] OR "Therapy"[MeSH Subheading] OR "Therapy"[All Fields] OR "Treatment"[All Fields] OR "treatment s"[All Fields]) AND "neoadjuvant radiation"[Title/Abstract])) 54656  #3 "immune checkpoint inhibitors"[Title/Abstract] OR "immune checkpoint blockers"[Title/Abstract] OR "checkpoint blockers immune"[Title/Abstract] OR "immune checkpoint inhibitor"[Title/Abstract] OR "checkpoint inhibitor immune"[Title/Abstract] OR "ctla 4 inhibitors"[Title/Abstract] OR "ctla 4 inhibitors"[Title/Abstract] OR "cytotoxic t lymphocyte associated protein 4 inhibitors"[Title/Abstract] OR "cytotoxic t lymphocyte associated protein 4 inhibitors"[Title/Abstract] OR "cytotoxic t lymphocyte associated protein 4 inhibitor"[Title/Abstract] OR "cytotoxic t lymphocyte associated protein 4 inhibitor"[Title/Abstract] OR "ctla 4 inhibitor"[Title/Abstract] OR "ctla 4 inhibitor"[Title/Abstract] OR "pd 1 inhibitors"[Title/Abstract] OR "pd 1 inhibitors"[Title/Abstract] OR "programmed cell death protein 1 inhibitor"[Title/Abstract] OR "programmed cell death protein 1 inhibitors"[Title/Abstract] OR "pd 1 inhibitor"[Title/Abstract] OR "inhibitor pd 1"[Title/Abstract] OR "pd 1 inhibitor"[Title/Abstract] OR "immune checkpoint blockade"[Title/Abstract] OR "checkpoint blockade immune"[Title/Abstract] OR "immune checkpoint inhibition"[Title/Abstract] OR "checkpoint inhibition immune"[Title/Abstract] OR "pd l1 inhibitors"[Title/Abstract] OR "pd l1 inhibitors"[Title/Abstract] OR "programmed death ligand 1 inhibitors"[Title/Abstract] OR "programmed death ligand 1 inhibitors"[Title/Abstract] OR "pd l1 inhibitor"[Title/Abstract] OR "pd l1 inhibitor"[Title/Abstract] OR "pd 1 pd l1 blockade"[Title/Abstract] OR "blockade pd 1 pd l1"[Title/Abstract] OR "pd 1 pd l1 blockade"[Title/Abstract] OR ("pembrolizumab"[Title/Abstract] OR "MK-3475"[Title/Abstract] OR "Keytruda"[Title/Abstract] OR "lambrolizumab"[Title/Abstract] OR "SCH-900475"[Title/Abstract] OR "atezolizumab"[Title/Abstract] OR ((((("immunoglobulin g"[Supplementary Concept] OR "immunoglobulin g"[All Fields] OR "immunoglobulin g1"[All Fields] OR "immunoglobulin g"[MeSH Terms] OR ("immunoglobulin"[All Fields] AND "g1"[All Fields])) AND ("antib technol j"[Journal] OR "anti"[All Fields])) AND (("human s"[All Fields] OR "humans"[MeSH Terms] OR "humans"[All Fields] OR "human"[All Fields]) AND ("cd274 protein human"[Supplementary Concept] OR "cd274 protein human"[All Fields] OR "cd antigen cd274"[All Fields]))) AND (("human s"[All Fields] OR "humans"[MeSH Terms] OR "humans"[All Fields] OR "human"[All Fields]) AND ("monoclonal"[All Fields] OR "monoclonality"[All Fields] OR "monoclonally"[All Fields] OR "monoclonals"[All Fields] OR "monoclone"[All Fields] OR "monoclones"[All Fields]) AND "heavy"[All Fields] AND ("chain"[All Fields] OR "chain s"[All Fields] OR "chains"[All Fields]))) AND ((("disulfides"[Supplementary Concept] OR "disulfides"[All Fields] OR "disulfide"[All Fields] OR "disulfides"[MeSH Terms] OR "disulphide"[All Fields] OR "disulphides"[All Fields]) AND ("human s"[All Fields] OR "humans"[MeSH Terms] OR "humans"[All Fields] OR "human"[All Fields]) AND ("monoclonal"[All Fields] OR "monoclonality"[All Fields] OR "monoclonally"[All Fields] OR "monoclonals"[All Fields] OR "monoclone"[All Fields] OR "monoclones"[All Fields])) AND "kappa chain dimer"[Title/Abstract])) OR "anti-PDL1"[Title/Abstract] OR "MPDL3280A"[Title/Abstract] OR "MPDL-3280A"[Title/Abstract] OR "Tecentriq"[Title/Abstract] OR "RG7446"[Title/Abstract] OR "RG-7446"[Title/Abstract] OR "durvalumab"[Title/Abstract] OR "MEDI4736"[Title/Abstract] OR "MEDI-4736"[Title/Abstract] OR "Imfinzi"[Title/Abstract]) 63055  #4 #1 and #2 and #3 304  #5 #4 and (clinicaltrial[Filter] OR randomizedcontrolledtrial[Filter]) 41 |
| **Web of Science** | #1 TS=("Neoadjuvant Therapies" OR "Therapy, Neoadjuvant" OR "Neoadjuvant Treatment" OR "Neoadjuvant Treatments" OR "Treatment, Neoadjuvant" OR "Neoadjuvant Chemotherapy" OR "Chemotherapy, Neoadjuvant" OR "Neoadjuvant Chemotherapies" OR "Neoadjuvant Chemotherapy Treatment" OR "Chemotherapy Treatment, Neoadjuvant" OR "Neoadjuvant Chemotherapy Treatments" OR "Treatment, Neoadjuvant Chemotherapy" OR "Neoadjuvant Chemoradiotherapy" OR "Chemoradiotherapy, Neoadjuvant" OR "Neoadjuvant Chemoradiotherapies" OR "Neoadjuvant Chemoradiation" OR "Chemoradiation, Neoadjuvant" OR "Neoadjuvant Chemoradiations" OR "Neoadjuvant Chemoradiation Therapy" OR "Chemoradiation Therapy, Neoadjuvant" OR "Neoadjuvant Chemoradiation Therapies" OR "Therapy, Neoadjuvant Chemoradiation" OR "Neoadjuvant Chemoradiation Treatment" OR "Chemoradiation Treatment, Neoadjuvant" OR "Neoadjuvant Chemoradiation Treatments" OR "Treatment, Neoadjuvant Chemoradiation" OR "Neoadjuvant Systemic Therapy" OR "Neoadjuvant Systemic Therapies" OR "Systemic Therapy, Neoadjuvant" OR "Therapy, Neoadjuvant Systemic" OR "Neoadjuvant Systemic Treatment" OR "Neoadjuvant Systemic Treatments" OR "Systemic Treatment, Neoadjuvant" OR "Treatment, Neoadjuvant Systemic" OR "Neoadjuvant Radiotherapy" OR "Neoadjuvant Radiotherapies" OR "Radiotherapy, Neoadjuvant" OR "Neoadjuvant Radiation" OR "Neoadjuvant Radiations" OR "Radiation, Neoadjuvant" OR "Neoadjuvant Radiation Therapy" OR "Neoadjuvant Radiation Therapies" OR "Radiation Therapy, Neoadjuvant" OR "Therapy, Neoadjuvant Radiation" OR "Neoadjuvant Radiation Treatment" OR "Neoadjuvant Radiation Treatments" OR "Radiation Treatment, Neoadjuvant" OR "Treatment, Neoadjuvant Radiation") 60856  #2 TS=("Triple Negative Breast Neoplasms" OR "ER-Negative PR-Negative HER2-Negative Breast Cancer" OR "ER Negative PR Negative HER2 Negative Breast Cancer" OR "ER-Negative PR-Negative HER2-Negative Breast Neoplasms" OR "ER Negative PR Negative HER2 Negative Breast Neoplasms" OR "Triple Negative Breast Cancer" OR "Triple-Negative Breast Cancer" OR "Breast Cancers, Triple-Negative" OR "Breast Cancer, Triple-Negative" OR "Triple-Negative Breast Cancers" OR "Triple-Negative Breast Neoplasm" OR "Breast Neoplasms, Triple-Negative" OR "Breast Neoplasm, Triple-Negative" OR "Triple Negative Breast Neoplasm" OR "Triple-Negative Breast Neoplasms") 32193  #3 TS=("Immune Checkpoint Blockers" OR "Checkpoint Blockers, Immune" OR "Immune Checkpoint Inhibitor" OR "Checkpoint Inhibitor, Immune" OR "CTLA-4 Inhibitors" OR "CTLA 4 Inhibitors" OR "Cytotoxic T-Lymphocyte-Associated Protein 4 Inhibitors" OR "Cytotoxic T Lymphocyte Associated Protein 4 Inhibitors" OR "Cytotoxic T-Lymphocyte-Associated Protein 4 Inhibitor" OR "Cytotoxic T Lymphocyte Associated Protein 4 Inhibitor" OR "CTLA-4 Inhibitor" OR "CTLA 4 Inhibitor" OR "PD-1 Inhibitors" OR "PD 1 Inhibitors" OR "Programmed Cell Death Protein 1 Inhibitor" OR "Programmed Cell Death Protein 1 Inhibitors" OR "PD-1 Inhibitor" OR "Inhibitor, PD-1" OR "PD 1 Inhibitor" OR "Immune Checkpoint Blockade" OR "Checkpoint Blockade, Immune" OR "Immune Checkpoint Inhibition" OR "Checkpoint Inhibition, Immune" OR "PD-L1 Inhibitors" OR "PD L1 Inhibitors" OR "Programmed Death-Ligand 1 Inhibitors" OR "Programmed Death Ligand 1 Inhibitors" OR "PD-L1 Inhibitor" OR "PD L1 Inhibitor" OR "PD-1-PD-L1 Blockade" OR "Blockade, PD-1-PD-L1" OR "PD 1 PD L1 Blockade" OR "durvalumab" OR "Imfinzi" OR "MEDI4736" OR "MEDI-4736" OR "camrelizumab" OR "SHR-1210" OR "SHR1210" OR "pembrolizumab" OR "lambrolizumab" OR "MK-3475" OR "MK 3475" OR "atezolizumab" OR "Tecentriq" OR "MPDL3280A" OR "MPDL-3280A" OR "RG7446") 38255  #4 #1 and #2 and #3 75 |
| **Cochrane** | #1 MeSH descriptor: [Triple Negative Breast Neoplasms] explode all trees 617  #2 "Triple-Negative Breast Neoplasms" or "ER Negative PR Negative HER2 Negative Breast Neoplasms" or "Triple Negative Breast Cancer" or "Breast Neoplasms, Triple-Negative" or "Breast Cancer, Triple-Negative" or "Triple-Negative Breast Cancers" or "ER-Negative PR-Negative HER2-Negative Breast Neoplasms" or "Triple-Negative Breast Cancer" or "Breast Cancers, Triple-Negative" or "Triple Negative Breast Neoplasm" or "ER-Negative PR-Negative HER2-Negative Breast Cancer" or "ER Negative PR Negative HER2 Negative Breast Cancer" or "Triple-Negative Breast Neoplasm" or "Breast Neoplasm, Triple-Negative" 2217  #3 #1 or #2 2217  #4 MeSH descriptor: [Neoadjuvant Therapy] explode all trees 2903  #5 "Systemic Treatment, Neoadjuvant" or "Neoadjuvant Systemic Therapy" or "Neoadjuvant Systemic Treatment" or "Systemic Therapy, Neoadjuvant" or "Neoadjuvant Systemic Treatments" or "Treatment, Neoadjuvant Systemic" or "Therapy, Neoadjuvant Systemic" or "Neoadjuvant Systemic Therapies" or "Neoadjuvant Radiation Treatment" or "Neoadjuvant Radiation" or "Neoadjuvant Radiation Therapies" or "Neoadjuvant Radiation Therapy" or "Neoadjuvant Radiations" or "Therapy, Neoadjuvant Radiation" or "Neoadjuvant Radiotherapy" or "Treatment, Neoadjuvant Radiation" or "Radiation Therapy, Neoadjuvant" or "Radiation, Neoadjuvant" or "Radiotherapy, Neoadjuvant" or "Neoadjuvant Radiation Treatments" or "Radiation Treatment, Neoadjuvant" or "Neoadjuvant Radiotherapies" or "Neoadjuvant Chemoradiation Treatments" or "Therapy, Neoadjuvant Chemoradiation" or "Chemoradiation Therapy, Neoadjuvant" or "Neoadjuvant Chemoradiations" or "Treatment, Neoadjuvant Chemoradiation" or "Neoadjuvant Chemoradiation Therapy" or "Chemoradiation Treatment, Neoadjuvant" or "Neoadjuvant Chemoradiation Treatment" or "Neoadjuvant Chemoradiation Therapies" or "Neoadjuvant Chemoradiotherapy" or "Neoadjuvant Chemoradiation" or "Neoadjuvant Chemoradiotherapies" or "Chemoradiotherapy, Neoadjuvant" or "Chemoradiation, Neoadjuvant" or "Neoadjuvant Chemotherapy Treatment" or "Treatment, Neoadjuvant Chemotherapy" or "Neoadjuvant Chemotherapy" or "Chemotherapy, Neoadjuvant" or "Neoadjuvant Chemotherapies" or "Chemotherapy Treatment, Neoadjuvant" or "Neoadjuvant Chemotherapy Treatments" or "Neoadjuvant Treatments" or "Neoadjuvant Therapies" or "Treatment, Neoadjuvant" or "Neoadjuvant Treatment" or "Therapy, Neoadjuvant" 9011  #6 #4 or #5 10123  #7 MeSH descriptor: [Immune Checkpoint Inhibitors] explode all trees 437  #8 "Cytotoxic T-Lymphocyte-Associated Protein 4 Inhibitor" or "Cytotoxic T Lymphocyte Associated Protein 4 Inhibitors" or "CTLA 4 Inhibitors" or "CTLA-4 Inhibitor" or "CTLA 4 Inhibitor" or "Cytotoxic T-Lymphocyte-Associated Protein 4 Inhibitors" or "CTLA-4 Inhibitors" or "Cytotoxic T Lymphocyte Associated Protein 4 Inhibitor" or "Programmed Cell Death Protein 1 Inhibitors" or "PD-1 Inhibitor" or "Inhibitor, PD-1" or "PD 1 Inhibitors" or "PD-1 Inhibitors" or "Programmed Cell Death Protein 1 Inhibitor" or "PD 1 Inhibitor" or "Checkpoint Inhibitors, Immune" or "Immune Checkpoint Inhibitor" or "Checkpoint Inhibitor, Immune" or "Immune Checkpoint Blockers" or "Checkpoint Blockers, Immune" or "PD 1 PD L1 Blockade" or "Blockade, PD-1-PD-L1" or "PD-1-PD-L1 Blockade" or "Immune Checkpoint Blockade" or "Checkpoint Blockade, Immune" or "Immune Checkpoint Inhibition" or "Checkpoint Inhibition, Immune" or "PD-L1 Inhibitors" or "PD L1 Inhibitors" or "Programmed Death-Ligand 1 Inhibitors" or "PD-L1 Inhibitor" or "Programmed Death Ligand 1 Inhibitors" or "PD L1 Inhibitor" or "pembrolizumab" or "lambrolizumab" or "MK-3475" or "Keytruda" or "nivolumab" or "Opdivo" or "BMS-936558" or "atezolizumab" or "Tecentriq" or "MPDL3280A" or "MPDL-3280A" or "durvalumab" or "Imfinzi" or "MEDI4736" or "MEDI-4736" or "camrelizumab" or "SHR-1210" 2652  #9 #7 or #8 2952  #10 #3 and #6 and #9 45 |
| **Embase** | #1 'triple negative breast cancer'/exp OR 'metastatic triple-negative breast cancer' OR 'tnbc' OR 'triple negative breast cancers' OR 'triple negative breast carcinoma' OR 'triple negative breast carcinomas' OR 'triple negative breast neoplasm' OR 'triple negative breast neoplasms' OR 'triple receptor negative breast cancer' OR 'triple receptor negative breast cancers' OR 'triple-negative bc' OR 'triple-negative metastatic breast cancer' OR 'triple-negative subset of breast cancer' OR 'triple negative breast cancer' 57031  #2 'neoadjuvant therapy'/exp OR 'neo-adjuvant drug therapy' OR 'neo-adjuvant drug treatment' OR 'neo-adjuvant medication' OR 'neo-adjuvant therapy' OR 'neo-adjuvant treatment' OR 'neoadjuvant drug therapy' OR 'neoadjuvant drug treatment' OR 'neoadjuvant medication' OR 'neoadjuvant treatment' OR 'neoadjuvant therapy' 97102  #3 'immune checkpoint inhibitor'/exp OR 'immune checkpoint blocker' OR 'immune checkpoint inhibitors' OR 'immune checkpoint inhibitor' OR 'pembrolizumab' OR 'atezolizumab' OR 'Durvalumab' OR 'Camrelizumab' 127264  #4 #1 AND #2 AND #3 1365  #5 #4 AND 'randomized controlled trial'/de 196 |

| **Table S3.** Baseline Characteristics of Studies Included in the Network Meta-Analysis | | | | | | | | | | |
| --- | --- | --- | --- | --- | --- | --- | --- | --- | --- | --- |
| Study  (Year; Design) | Registration  (NCT) | Randomization Ratio  (Exp: Ctrl) | Sample Size  (Exp / Ctrl) | Stage | Intervention Arm(s) | Control Arm | pCR Definition | PD-L1  Assay / Cut-off | Median Follow-up | Outcomes Reported |
| I-SPY2^[18]^  (2020; OL) | NCT01042379 | ≈1:4 | 29 / 181 | II–III | **Pembro-NA**: Pembro 200 mg IV q3w × 4 → weekly PTX × 12 → AC × 4 | Weekly PTX × 12 → AC × 4 | ypT0/Tis ypN0 | Not restricted | 2.8 y | pCR |
| GeparNuevo^[16]^  (2022; DB) | NCT02685059 | 1:1 | 88 / 86 | cT1b–cT4a-d | **Durva-NA**: Window: Durva 750 mg × 1; NA: Durva 1.5 g q4w + nab-PTX 125 mg/m² qw × 12 → Durva 1.5 g q4w + EC × 4; No adjuvant ICI | Window: Placebo × 1; same chemo; No adjuvant ICI | ypT0 ypN0 | IC ≥1% | 43 m | pCR, OS |
| NCI-10013^[20]^  (2022; OL) | NCT02883062 | 2:1 | 45 / 16 | II–III | **Atezo-NA**: Atezo 1200 mg q3w × 4 + Carbo AUC5 q3w × 4 + PTX 80 mg/m² qw × 12 → ddAC × 4; No adjuvant ICI | Carbo + PTX → ddAC; No ICI | ypT0/Tis ypN0 | IC ≥1% | 6.6 m | pCR, AEs ≥3 |
| NeoTRIP^[21]^  (2022; OL) | NCT02620280 | 1:1 | 138 / 142 | - | **Atezo-NA**: Atezo 1200 mg d1 + Carbo AUC2 d1/8 + nab-PTX 125 mg/m² d1/8, q28d × 3 | Carbo AUC2 d1/8 + nab-PTX 125 mg/m² d1/8, q28d × 3 | ypT0 ypN0 | IC ≥1% | 54 m | pCR, AEs ≥3 |
| PLANeT^[22]^  (2025; OL) | - | 1:1 | 78 / 79 | II–III | **Pembro-NA-low**: Pembro 50 mg IV q6w × 3 + ddAC × 4 → PTX × 4 | ddAC × 4 → PTX × 4 | ypT0 ypN0 | Not restricted | Time to surgery | pCR, AEs ≥3 |
| IMpassion031^[17]^ (2024; DB) | NCT03197935 | 1:1 | 165 / 168 | II–III | **Atezo-P**: NA: Atezo 840 mg q2w × 12 + nab-PTX 125 mg/m² qw × 12 → ddAC × 4; Adj: Atezo 1200 mg q3w × 11 | NA: Placebo q2w × 12 + same chemo; Adj: Placebo q3w × 11 | ypT0/Tis ypN0 | IC ≥1% | 40.3 m (EFS) | pCR, EFS, OS, AEs ≥3 |
| KEYNOTE-522^[19]^  (2024; DB) | NCT03036488 | 2:1 | 784 / 390 | II–III | **Pembro-P**: NA: Pembro 200 mg q3w × 8 + PTX 80 mg/m² qw × 12 + Carbo AUC5 q3w × 4 → AC × 4; Adj: Pembro 200 mg q3w × 9 | NA: Placebo q3w × 8 + same chemo; Adj: Placebo q3w × 9 | ypT0/Tis ypN0 | CPS ≥1 | 39.1 m(EFS)  75m (OS) | pCR, EFS, OS |
| CamRelief^[15]^  (2024; DB) | NCT04613674 | 1:1 | 222 / 219 | II–III | **Camre-P**: Cycles 1–16: Camre 200 mg q2w + nab-PTX 100 mg/m² d1,8,15 + Carbo AUC1.5 d1,8,15; Cycles 17–24: Camre 200 mg q2w + EC; Adj: Camre 200 mg q2w up to 1 y | Placebo q2w + same chemo; No adjuvant ICI | ypT0/Tis ypN0 | CPS <10 vs ≥10 | 14.4 m | pCR, AEs ≥3 |

Abbreviations:

db = double‑blind; Exp. = experimental; Ctrl. = control; NA = neoadjuvant only; P = perioperative (neoadj+adjuv); Pembro = pembrolizumab; Durva = durvalumab; Atezo = atezolizumab; Camre = camrelizumab; PTX = paclitaxel; AC = doxorubicin/cyclophosphamide; EC = epirubicin/cyclophosphamide; dd = dose‑dense; Carbo = carboplatin; AUC = area under concentration‑time curve; qw = weekly; q2w = every 2 weeks; q3w = every 3 weeks; q6w = every 6 weeks; NACT = neoadjuvant chemotherapy; ICI = immune checkpoint inhibitor;pCR, pathological complete response.

**Table S4.Pairwise Meta-Analysis of Pathological Complete Response: Individual Study Data and Pooled Odds Ratios**

| **Study** | **Events/Total, Experimental Group** | **Events/Total, Control Group** | **OR (95% CI)** |
| --- | --- | --- | --- |
| I-SPY2 | 17/29 | 40/181 | 2.96 (1.47–5.97) |
| GeparNuevo | 47/88 | 38/86 | 1.44 (0.81–2.55) |
| NCI-10013 | 25/45 | 3/16 | 5.50 (1.30–23.3) |
| NeoTRIP | 67/138 | 63/142 | 1.18 (0.74–1.89) |
| PLANeT Trial | 42/78 | 32/79 | 1.68 (0.92–3.07) |
| IMpassion031 | 96/165 | 69/168 | 2.05 (1.37–3.06) |
| KEYNOTE-522 | 508/784 | 200/390 | 1.81 (1.44–2.27) |
| CamRelief | 126/222 | 98/219 | 1.63 (1.14–2.33) |

**Table S5.Pairwise Meta-Analysis of Overall Survival: Individual Study Hazard Ratios**

| **Study** | **HR** | **Lower 95% CI** | **Upper 95% CI** |
| --- | --- | --- | --- |
| GeparNuevo | 0.24 | 0.08 | 0.72 |
| IMpassion031 | 0.56 | 0.30 | 1.04 |
| KEYNOTE‑522 | 0.63 | 0.48 | 0.82 |

**Table S6.Pairwise Meta-Analysis of Grade ≥3 Adverse Events: Individual Study Data and Pooled Odds Ratios**

| **Study** | **Events/Total, Experimental** | **Events/Total, Control** | **OR (95% CI)** |
| --- | --- | --- | --- |
| NCI-10013 | 26/45 | 10/16 | 0.93 (0.60–1.43) |
| NeoTRIP | 107/138 | 98/140 | 1.11 (0.95–1.29) |
| PLANeT Trial | 42/74 | 32/78 | 1.38 (0.98–1.96) |
| IMpassion031 | 93/164 | 89/167 | 1.06 (0.87–1.30) |
| CamRelief | 198/222 | 182/219 | 1.07 (0.98–1.18) |

| **Table S7. SUCRA-Based Ranking of Treatment Regimens for Endpoints in the Bayesian Network Meta-Analysis(Analysis by Target Class)** | | | | | | |
| --- | --- | --- | --- | --- | --- | --- |
| Treatment | OS | | PCR | | AEs≥3 | |
|  | SUCRA(%) | Rank | SUCRA(%) | Rank | SUCRA(%) | Rank |
| CT | 13 | 4 | 7.7 | 5 | 80.4 | 1 |
| PD-L1(NA)+CT | 95.1 | 1 | 48.1 | 3 | 53.2 | 3 |
| PD-L1(P)+CT | 56.4 | 2 | 61.6 | 2 | 62.7 | 2 |
| PD-1(P)+CT | 47.1 | 3 | 50.2 | 4 | 30.6 | 4 |
| PD-1(NA)+CT | - |  | 82.4 | 1 | 23.1 | 5 |

| **Table S8. SUCRA-Based Ranking of Treatment Regimens for Endpoints in the Bayesian Network Meta-Analysis(Analysis by Specific Agent)** | | | | | | |
| --- | --- | --- | --- | --- | --- | --- |
| Treatment | OS | | PCR | | AEs≥3 | |
|  | SUCRA(%) | Rank | SUCRA(%) | Rank | SUCRA(%) | Rank |
| CT | 13 | 4 | 21.0 | 7 | 80.3 | 1 |
| Durva-(NA)+CT | 95.1 | 1 | 43.0 | 6 | - | - |
| Atezo-(P)+CT | 56.4 | 2 | 56.5 | 3 | 62.5 | 2 |
| Pembro-(P)+CT | 47.1 | 3 | 50.8 | 4 | - | - |
| Atezo-(NA)+CT | - | - | 58.3 | 2 | 52.9 | 3 |
| Camre-(P)+CT | - | - | 47.6 | 5 | 30.7 | 4 |
| Pembro-(NA)+CT | - | - | 72.2 | 1 | 23.4 | 5 |

| **Table S9. Model Fit Statistics for Consistency and Inconsistency Models in Bayesian Network Meta-Analysis by Target Class** | | | | | | |
| --- | --- | --- | --- | --- | --- | --- |
| Outcome Measure |  | Model Type | Dbar | pD | DIC | I2 |
| OS |  | Consistency | 2.988589 | 2.988418 | 5.977007 | 33 |
|  |  | Inconsistency | 2.989363 | 2.989296 | 5.978659 | 33 |
| pCR |  | Consistency | 19.51134 | 16.38355 | 35.89489 | 13 |
|  |  | Inconsistency | 20.08441 | 16.10070 | 36.18511 | 15 |
| AE≥3 |  | Consistency | 9.783372 | 9.372720 | 19.156092 | 8 |
|  |  | Inconsistency | 9.586076 | 9.249763 | 18.835839 | 6 |

| **Table S10. Model Fit Statistics for Consistency and Inconsistency Models in Bayesian Network Meta-Analysis by Specific Agent** | | | | | | |
| --- | --- | --- | --- | --- | --- | --- |
| Outcome Measure |  | Model Type | Dbar | pD | DIC | I2 |
| OS |  | Consistency | 3.008343 | 3.008075 | 6.016418 | 34 |
|  |  | Inconsistency | 2.988811 | 2.988701 | 5.977512 | 33 |
| pCR |  | Consistency | 18.58187 | 17.54747 | 36.12934 | 9 |
|  |  | Inconsistency | 18.45947 | 17.39417 | 35.85363 | 8 |
| AE≥3 |  | Consistency | 9.783372 | 9.372720 | 19.156092 | 8 |
|  |  | Inconsistency | 9.586076 | 9.249763 | 18.835839 | 6 |

| **Table S11. Model Fit Statistics for Fixed‑Effect and Random‑Effect Bayesian Network Meta‑Analysis by Target Class** | | | | | | |
| --- | --- | --- | --- | --- | --- | --- |
| Outcome Measure |  | Model Type | Dbar | pD | DIC |  |
| OS |  | Random ffects | 3.21 | 4.24 | 7.45 |  |
|  |  | Fixed Effects | 3.24 | 4.18 | 7.42 |  |
| pCR |  | Random Effects | 20.09 | 16.16 | 36.34 |  |
|  |  | Fixed Effects | 24.03 | 12.97 | 37.00 |  |
| AE≥3 |  | Random Effects | 9.57 | 9.22 | 18.79 |  |
|  |  | Fixed Effects | 9.61 | 8.91 | 18.52 |  |

| **Table S12. Model Fit Statistics for Fixed‑Effect and Random‑Effect Bayesian Network Meta‑Analysis by Specific Agent** | | | | | | |
| --- | --- | --- | --- | --- | --- | --- |
| Outcome Measure |  | Model Type | Dbar | pD | DIC |  |
| OS |  | Random Effects | 3.05 | 3.12 | 6.17 |  |
|  |  | Fixed Effects | 3.11 | 3.22 | 6.33 |  |
| pCR |  | Random Effects | 18.39 | 17.35 | 35.73 |  |
|  |  | Fixed Effects | 25.72 | 14.95 | 40.67 |  |
| AE≥3 |  | Random Effects | 9.57 | 9.22 | 18.79 |  |
|  |  | Fixed Effects | 9.61 | 8.91 | 18.52 |  |

**Table S13.CINeMA (Confidence in Network Meta-Analysis) assessment of pathological complete response (pCR) for comparisons between PD-1 and PD-L1 inhibitors combined with chemotherapy versus chemotherapy alone or versus each other.**

| **Comparison** | **Number of studies** | **Within-study bias** | **Reporting bias** | **Indirectness** | **Imprecision** | **Heterogeneity** | **Incoherence** | **Confidence rating** | **Reason(s) for downgrading** |
| --- | --- | --- | --- | --- | --- | --- | --- | --- | --- |
| CT:PD-1(NA)+  CT | 2 | Some concerns | Low risk | No concerns | No concerns | Some concerns | Some concerns | Very low | [] |
| CT:PD-1(P)+  CT | 2 | No concerns | Low risk | No concerns | Some concerns | No concerns | Some concerns | Low | [] |
| CT:PD-L1(NA)+  CT | 4 | Some concerns | Low risk | No concerns | No concerns | No concerns | Some concerns | Low | [] |
| CT:PD-L1(P)+  CT | 1 | No concerns | Low risk | No concerns | No concerns | No concerns | Some concerns | Moderate | [] |
| PD-1(NA)+CT:  PD-1(P)+CT | 0 | Some concerns | Low risk | No concerns | Some concerns | No concerns | Some concerns | Very low | [] |
| PD-1(NA)+CT:  PD-L1(NA)+CT | 0 | Some concerns | Low risk | No concerns | No concerns | No concerns | Some concerns | Low | [] |
| PD-1(NA)+CT:  PD-L1(P)+CT | 0 | No concerns | Low risk | No concerns | Some concerns | No concerns | Some concerns | Low | [] |
| PD-1(P)+CT:  PD-L1(NA)+CT | 0 | Some concerns | Low risk | No concerns | No concerns | No concerns | Some concerns | Low | [] |
| PD-1(P)+CT:  PD-L1(P)+CT | 0 | No concerns | Low risk | No concerns | Some concerns | No concerns | Some concerns | Low | [] |
| PD-L1(NA)+CT:  PD-L1(P)+CT | 0 | No concerns | Low risk | No concerns | Some concerns | No concerns | Some concerns | Low | [] |
| CT:PD-1(NA)+  CT | 2 | Some concerns | Low risk | No concerns | No concerns | Some concerns | Some concerns | Very low | [] |
| CT:PD-1(P)+  CT | 2 | No concerns | Low risk | No concerns | Some concerns | No concerns | Some concerns | Low | [] |
| CT:  PD-L1(NA)+CT | 4 | Some concerns | Low risk | No concerns | No concerns | No concerns | Some concerns | Low | [] |
| CT:  PD-L1(P)+CT | 1 | No concerns | Low risk | No concerns | No concerns | No concerns | Some concerns | Moderate | [] |
| PD-1(NA)+CT:  PD-1(P)+CT | 0 | Some concerns | Low risk | No concerns | Some concerns | No concerns | Some concerns | Very low | [] |

**Table S14.CINeMA assessment of adverse events (AEs) for class-level comparisons between PD-1 and PD-L1 inhibitors combined with chemotherapy versus chemotherapy alone or versus each other.**

| **Comparison** | **Number of studies** | **Within-study bias** | **Reporting bias** | **Indirectness** | **Imprecision** | **Heterogeneity** | **Incoherence** | **Confidence rating** | **Reason(s) for downgrading** |
| --- | --- | --- | --- | --- | --- | --- | --- | --- | --- |
| CT:PD-1(NA)+CT | 1 | Some concerns | Low risk | No concerns | No concerns | Some concerns | Some concerns | Very low | [] |
| CT:PD-1(P)+CT | 1 | Some concerns | Low risk | No concerns | No concerns | No concerns | Some concerns | Low | [] |
| CT:PD-L1(NA)+CT | 2 | Some concerns | Low risk | No concerns | No concerns | No concerns | Some concerns | Low | [] |
| CT:PD-L1(P)+CT | 1 | No concerns | Low risk | No concerns | Some concerns | No concerns | Some concerns | Low | [] |
| PD-1(NA)+CT:PD-1(P)+CT | 0 | Some concerns | Low risk | No concerns | Some concerns | No concerns | Some concerns | Very low | [] |
| PD-1(NA)+CT:PD-L1(NA)+CT | 0 | Some concerns | Low risk | No concerns | No concerns | Some concerns | Some concerns | Very low | [] |
| PD-1(NA)+CT:PD-L1(P)+CT | 0 | No concerns | Low risk | No concerns | No concerns | Some concerns | Some concerns | Low | [] |
| PD-1(P)+CT:PD-L1(NA)+CT | 0 | Some concerns | Low risk | No concerns | No concerns | No concerns | Some concerns | Low | [] |
| PD-1(P)+CT:PD-L1(P)+CT | 0 | No concerns | Low risk | No concerns | No concerns | No concerns | Some concerns | Moderate | [] |
| PD-L1(NA)+CT:PD-L1(P)+CT | 0 | No concerns | Low risk | No concerns | No concerns | Some concerns | Some concerns | Low | [] |

**Table S15.CINeMA assessment of pathological complete response (pCR) for agent-specific comparisons of immune checkpoint inhibitors combined with chemotherapy versus chemotherapy alone or versus each other.**

| **Comparison** | **Number of studies** | **Within-study bias** | **Reporting bias** | **Indirectness** | **Imprecision** | **Heterogeneity** | **Incoherence** | **Confidence rating** | **Reason(s) for downgrading** |
| --- | --- | --- | --- | --- | --- | --- | --- | --- | --- |
| Atezo-(NA)+CT:CT | 2 | Some concerns | Low risk | No concerns | No concerns | No concerns | Some concerns | Low | [] |
| Atezo-(P)+CT:CT | 1 | No concerns | Low risk | No concerns | No concerns | No concerns | Some concerns | Moderate | [] |
| Camre-(P)+CT:CT | 1 | Some concerns | Low risk | No concerns | No concerns | No concerns | Some concerns | Low | [] |
| CT:Durva-(NA)+CT | 2 | No concerns | Low risk | No concerns | No concerns | No concerns | Some concerns | Moderate | [] |
| CT:Pembro-(NA)+CT | 2 | Some concerns | Low risk | No concerns | No concerns | No concerns | Some concerns | Low | [] |
| CT:Pembro-(P)+CT | 1 | No concerns | Low risk | No concerns | Some concerns | No concerns | Some concerns | Low | [] |
| Atezo-(NA)+CT:Atezo-(P)+CT | 0 | No concerns | Low risk | No concerns | Some concerns | No concerns | Some concerns | Low | [] |
| Atezo-(NA)+CT:Camre-(P)+CT | 0 | Some concerns | Low risk | No concerns | No concerns | No concerns | Some concerns | Low | [] |
| Atezo-(NA)+CT:Durva-(NA)+CT | 0 | Some concerns | Low risk | No concerns | Some concerns | No concerns | Some concerns | Very low | [] |
| Atezo-(NA)+CT:Pembro-(NA)+CT | 0 | Some concerns | Low risk | No concerns | No concerns | No concerns | Some concerns | Low | [] |
| Atezo-(NA)+CT:Pembro-(P)+CT | 0 | No concerns | Low risk | No concerns | Some concerns | No concerns | Some concerns | Low | [] |
| Atezo-(P)+CT:Camre-(P)+CT | 0 | No concerns | Low risk | No concerns | No concerns | No concerns | Some concerns | Moderate | [] |
| Atezo-(P)+CT:Durva-(NA)+CT | 0 | No concerns | Low risk | No concerns | Some concerns | No concerns | Some concerns | Low | [] |
| Atezo-(P)+CT:Pembro-(NA)+CT | 0 | No concerns | Low risk | No concerns | Some concerns | No concerns | Some concerns | Low | [] |
| Atezo-(P)+CT:Pembro-(P)+CT | 0 | No concerns | Low risk | No concerns | No concerns | No concerns | Some concerns | Moderate | [] |
| Camre-(P)+CT:Durva-(NA)+CT | 0 | Some concerns | Low risk | No concerns | No concerns | No concerns | Some concerns | Low | [] |
| Camre-(P)+CT:Pembro-(NA)+CT | 0 | Some concerns | Low risk | No concerns | Some concerns | No concerns | Some concerns | Very low | [] |
| Camre-(P)+CT:Pembro-(P)+CT | 0 | No concerns | Low risk | No concerns | Some concerns | No concerns | Some concerns | Low | [] |
| Durva-(NA)+CT:Pembro-(NA)+CT | 0 | Some concerns | Low risk | No concerns | Some concerns | No concerns | Some concerns | Very low | [] |
| Durva-(NA)+CT:Pembro-(P)+CT | 0 | No concerns | Low risk | No concerns | Some concerns | No concerns | Some concerns | Low | [] |
| Pembro-(NA)+CT:Pembro-(P)+CT | 0 | No concerns | Low risk | No concerns | Some concerns | No concerns | Some concerns | Low | [] |

**Table S16.CINeMA assessment of adverse events (AEs) for agent-specific comparisons of immune checkpoint inhibitors combined with chemotherapy versus chemotherapy alone or versus each other.**

| **Comparison** | **Number of studies** | **Within-study bias** | **Reporting bias** | **Indirectness** | **Imprecision** | **Heterogeneity** | **Incoherence** | **Confidence rating** | **Reason(s) for downgrading** |
| --- | --- | --- | --- | --- | --- | --- | --- | --- | --- |
| Atezo-(NA)+CT:CT | 2 | Some concerns | Low risk | No concerns | No concerns | Some concerns | Some concerns | Very low | [] |
| Atezo-(P)+CT:CT | 1 | No concerns | Low risk | No concerns | Some concerns | No concerns | Some concerns | Low | [] |
| Camre-(P)+CT:CT | 1 | Some concerns | Low risk | No concerns | No concerns | Some concerns | Some concerns | Very low | [] |
| CT:Pembro-(NA)+CT | 1 | Some concerns | Low risk | No concerns | No concerns | No concerns | Some concerns | Low | [] |
| Atezo-(NA)+CT:Atezo-(P)+CT | 0 | No concerns | Low risk | No concerns | Some concerns | No concerns | Some concerns | Low | [] |
| Atezo-(NA)+CT:Camre-(P)+CT | 0 | Some concerns | Low risk | No concerns | No concerns | No concerns | Some concerns | Low | [] |
| Atezo-(NA)+CT:Pembro-(NA)+CT | 0 | Some concerns | Low risk | No concerns | No concerns | No concerns | Some concerns | Low | [] |
| Atezo-(P)+CT:Camre-(P)+CT | 0 | No concerns | Low risk | No concerns | Some concerns | No concerns | Some concerns | Low | [] |
| Atezo-(P)+CT:Pembro-(NA)+CT | 0 | No concerns | Low risk | No concerns | No concerns | No concerns | Some concerns | Moderate | [] |
| Camre-(P)+CT:Pembro-(NA)+CT | 0 | Some concerns | Low risk | No concerns | Some concerns | No concerns | Some concerns | Very low | [] |

**Figure S1.** MCMC Convergence Diagnostics for Overall Survival Stratified by Target Class.

**Figure S2.** MCMC Convergence Diagnostics for Overall Survival Stratified by Specific Agent.

**Figure S3.** MCMC Convergence Diagnostics for Pathological Complete Response Stratified by Target Class.

**Figure S4.** MCMC Convergence Diagnostics for Pathological Complete Response Stratified by Specific Agent.

**Figure S5.** MCMC Convergence Diagnostics for Grade ≥3 Adverse Events Stratified by Target Class.

**Figure S6.** MCMC Convergence Diagnostics for Grade ≥3 Adverse Events Stratified by Specific Agent.

**Figure S7.** Trajectory Curves and Density Plots of Overall Survival According to Immune Checkpoint Inhibitor Target Class.

**Figure S8.** Trajectory Curves and Density Plots of Overall Survival According to Specific Immune Checkpoint Inhibitor Agents.

**Figure S9.** Trajectory Curves and Density Plots of Pathological Complete Response According to Immune Checkpoint Inhibitor Target Class.

**Figure S10.** Trajectory Curves and Density Plots of Pathological Complete Response According to Specific Immune Checkpoint Inhibitor Agents.

**Figure S11.** Trajectory Curves and Density Plots of Grade ≥3 Adverse Events According to Immune Checkpoint Inhibitor Target Class.

**Figure S12.** Trajectory Curves and Density Plots of Grade ≥3 Adverse Events According to Specific Immune Checkpoint Inhibitor Agents.

**Figure S13.** Rank Probability Heatmap of Overall Survival Outcomes Stratified by Target Class.

**Figure S14.** Rank Probability Heatmap of Overall Survival Outcomes Stratified by Specific Agent.

**Figure S15.** Rank Probability Heatmap of Pathological Complete Response Outcomes Stratified by Target Class.

**Figure S16.** Rank Probability Heatmap of Pathological Complete Response Outcomes Stratified by Specific Agent.

**Figure S17. Rank Probability Heatmap of Grade ≥3 Adverse Events Outcomes Stratified by Target Class** .

**Figure S18.** Rank Probability Heatmap of Grade ≥3 Adverse Events Outcomes Stratified by Specific Agent.

**Figure S19.** Funnel Plots Illustrating Pathological Complete Response Rates According to Immune Checkpoint Inhibitor Target Class.

**Figure S20.** Funnel Plots Illustrating Pathological Complete Response Rates According to Specific Immune Checkpoint Inhibitor Agents.

**Figure S21.** Sensitivity Analysis League Table for Pathological Complete Response and Grade ≥3 Adverse Events (Excluding PLANeT Trial).
